# Predicting Adsorbate-Solvent Interaction Energies in Zeolite Pores Using Convolutional Neural Networks with Attention Mechanisms

**DOI:** 10.1021/acs.jcim.6c02080

**Published:** 2026-09-30

**Authors:** Jiexin Shi, Xiuting Chen, Rachel B. Getman

**Affiliations:** William G. Lowrie Department of Chemical and Biomolecular Engineering, 2647 The Ohio State University, Columbus, Ohio 43210, United States

## Abstract

Solvent effects play a critical role in determining reaction thermodynamics and kinetics in liquid-phase heterogeneous catalysis, particularly in confined nanoporous environments such as zeolite pores. However, accurate prediction of solvation thermodynamics remains computationally prohibitive due to the need for extensive first-principles calculations on diverse solvent configurations. In this work, we present an attention-enhanced three-dimensional convolutional neural network (3D-CNN) framework to predict solvation interaction energies (Δ*E_int_*) of adsorbates in Ti-faujasite (Ti-FAU) zeolite pores. Molecular dynamics (MD) snapshots of C_1_–C_3_ oxygenates in aqueous and water–methanol solvent environments are transformed into multichannel voxel grid representations that encode spatially resolved atomistic information. The 3D-CNN architecture incorporates separate adsorbate and solvent branches and employs convolutional block attention modules to dynamically prioritize spatial regions and feature channels relevant to adsorbate–solvent interactions. Trained on density functional theory (DFT)-derived interaction energies from a multiscale modeling workflow, the model achieves a mean absolute error of 0.08 eV for individual configurations and 0.04 eV for ensemble-averaged solvation energies, approaching DFT accuracy while substantially reducing computational cost. Cross-validation (CV) using simulation-system, solvent-composition, and pore-type splits demonstrates predictive performance on held-out systems within the represented chemical domain and across the solvent and pore conditions considered in this study. Analysis of spatial attention maps and feature attributions reveals that the model emphasizes adsorbate-proximal solvent structures and hydrogen-bonding interactions, consistent with physical intuition. This framework enables efficient and interpretable prediction of solvation interaction energies in confined systems and provides a scalable pathway for mapping structural information sampled from force-field-MD to DFT targets.

## Graphical abstract

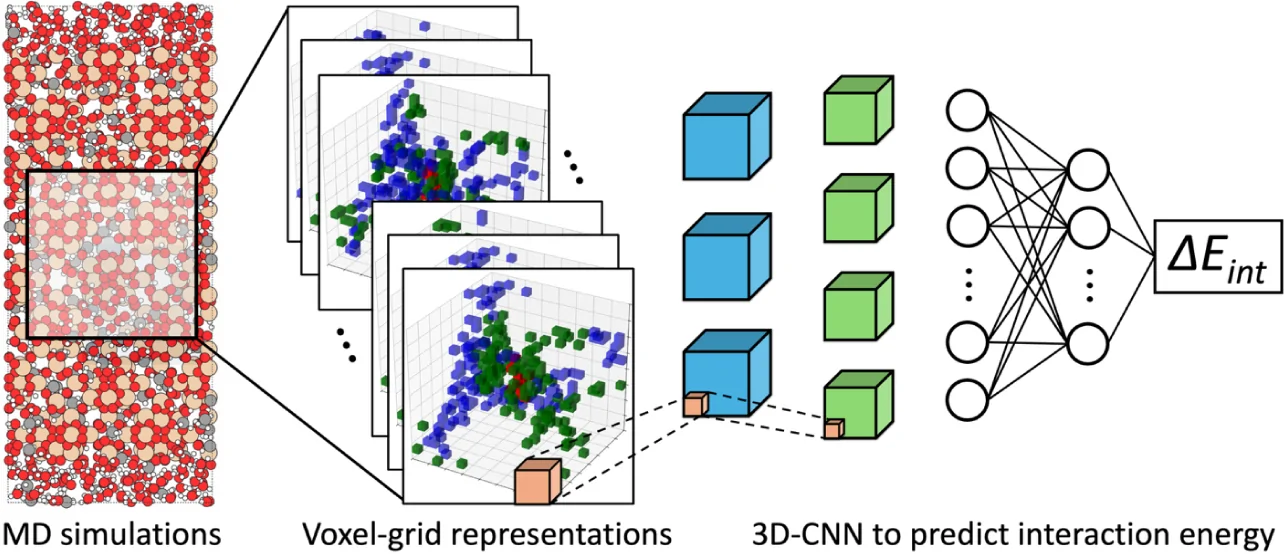

## 1 Introduction

Liquid-phase heterogeneous catalysis is relevant to a variety of industrially important processes, such as biomass conversion, hydrocarbon upgrading, and electrocatalysis.^1–4^ In these systems, solvents play a pivotal role by influencing reaction kinetics, selectivity, and thermodynamics,^5,6^ for example, through local solvent organization and hydrogen-bonding networks that stabilize reactive intermediates and influence activation barriers.^7–9^ These solvent effects are particularly pronounced in confined nanoporous environments such as zeolite pores, where spatial confinement can substantially alter solvation behavior and thereby affect reaction thermodynamics, kinetics, and mechanisms.^5,10^ Specifically, such confinement induces a variety of local solvent structures, ranging from isolated gas-like clusters to condensed liquid-like networks, including ordered interfacial layers and hydrogen-bonded chains of solvent molecules.^11,12^ These distinct solvent structures influence the strength of adsorbate–solvent interactions and contribute to the broader solvent effects governing macroscopic catalytic phenomena. Consequently, obtaining accurate solvation thermodynamic properties, particularly the solvation free energies of adsorbates at solid–liquid interfaces, is essential for understanding catalytic activity and selectivity in these systems.

Achieving this goal requires methodologies that balance computational efficiency and accuracy. This is particularly challenging in confined environments, where local solvent structures can differ substantially from those in bulk liquids. For example, implicit-solvent models may be inadequate because they primarily represent bulk dielectric and cavity effects and do not explicitly capture the diverse solvent structures induced by confinement.^12^ Thus, solvation thermodynamic properties are conventionally computed using density functional theory (DFT) with explicit solvent configurations.^13–15^ However, the chemical and structural complexity of confined catalytic systems makes such first-principles calculations impractical. Further, typical DFT workflows rely on human-generated rather than physics-generated solvent structures and hence are susceptible to bias. Whereas *ab initio* molecular dynamics (AIMD) could in theory resolve this issue, it is too computationally intensive to adequately sample solvent structure.^16^ Multiscale approaches that combine classical MD with quantum mechanics (QM) calculations are therefore needed, but they remain computationally demanding, which limits large-scale screening.^12,17^ One method for reducing this computational expense is to use machine learning (ML) to predict thermodynamic properties from sampled configurations. However, traditional ML approaches often rely on hand-crafted descriptors, which present significant challenges in nanoporous environments. Specifically, when applied to confined spaces, conventional spatial descriptors can be difficult to define and may inadequately represent the complex three-dimensional arrangements of solvent molecules within porous frameworks.

Deep learning is a promising alternative. In recent years, deep learning methods have been increasingly applied to learn structure–property relationships.^18–20^ Inspired by computer vision techniques that handle three-dimensional object recognition and spatial data processing,^21^ molecular configurations from MD simulations can be transformed into voxel grids, which are volumetric representations that discretize three-dimensional space into cubic elements. When combined with 3D convolutional neural networks (3D-CNNs), this voxel-based approach has demonstrated success in predicting binding affinities,^22–28^ formation energies,^29^ and reaction rates.^30^ Because they can learn hierarchical spatial patterns from atomic configurations, 3D-CNNs have the potential to capture the spatial patterns that govern adsorbate–solvent interactions. Further, voxel-grid representations explicitly resolve three-dimensional structural features rather than aggregating them into lower-dimensional descriptors, making them well suited to model the diverse and spatially heterogeneous solvent networks that arise in confined spaces.

As a step toward more accurate and efficient calculations of solvent effects in confined spaces, in this work, we develop a voxel-based 3D-CNN framework to predict solvation interaction energies (Δ*E_int_*), which are the energies of interaction between a solvent and an adsorbate, in zeolite pores. We specifically compute solvation interaction energies for species adsorbed within Ti-faujasite (Ti-FAU) zeolite. The 3D-CNN model is trained on energies calculated with our group’s multiscale sampling (MSS) method, which integrates classical MD with DFT.^31^ Specifically, solvent molecule configurations are sampled using classical MD, and the adsorbate–solvent interaction energies are calculated using DFT. These interaction energies constitute an energetic contribution within the broader MSS evaluation of solvation thermodynamics but are not complete solvation free energies. Calculation of Δ*E*_int_ using DFT is the most computationally intensive step in the MSS workflow. Our data set comprises MD snapshots of C_1_–C_3_ oxygenates in both hydrophilic and hydrophobic pores within Ti-FAU zeolites. MD snapshots are transformed into multichannel voxel grids that encode adsorbate–solvent systems using atomistic properties such as mass, partial charge, and Lennard–Jones parameters, thereby avoiding the need for system-level molecular descriptors or fingerprints. This encoding enables the model to automatically learn the spatial patterns of solvent structuring. Our approach achieves a mean absolute error (MAE) of 0.08 eV for predictions of individual snapshots and 0.04 eV for the ensemble-averaged solvation interaction energies (Δ*E_sol_*), achieving accuracy comparable to DFT calculations. The trained model hence serves as an efficient surrogate for DFT energy calculations within the MSS workflow. To further enhance the model’s ability to discern chemically relevant representations, we integrate attention mechanisms into the 3D-CNN architecture. The convolutional block attention modules dynamically reweight learned feature channels and spatial locations to prioritize informative solvent structures around the adsorbate. Consequently, the resulting attention maps provide insight into the spatial regions that the model identifies as most relevant for predicting Δ*E_int_*. This work demonstrates that deep learning can accelerate the evaluation of Δ*E_int_* in heterogeneous catalysis by providing an efficient surrogate for DFT interaction energy calculations within the MSS workflow, thereby offering a scalable pathway toward high-throughput screening of solvent effects in confined catalytic environments.

## 2 Methods

The overall workflow of this research is illustrated in Figure 1. First, an ensemble of snapshots capturing diverse solvent configurations around an adsorbate is generated with MD. Values of Δ*E_int_* are then calculated for each snapshot using DFT, and the adsorbate–solvent configurations within each snapshot are transformed into multichannel voxel grids. The voxelized representations are then provided as inputs to a 3D-CNN. The network is trained to predict $\Delta E_{int}^{ML}$ for each snapshot, and its performance is evaluated against $\Delta E_{int}^{DFT}$ .

**Figure 1:**
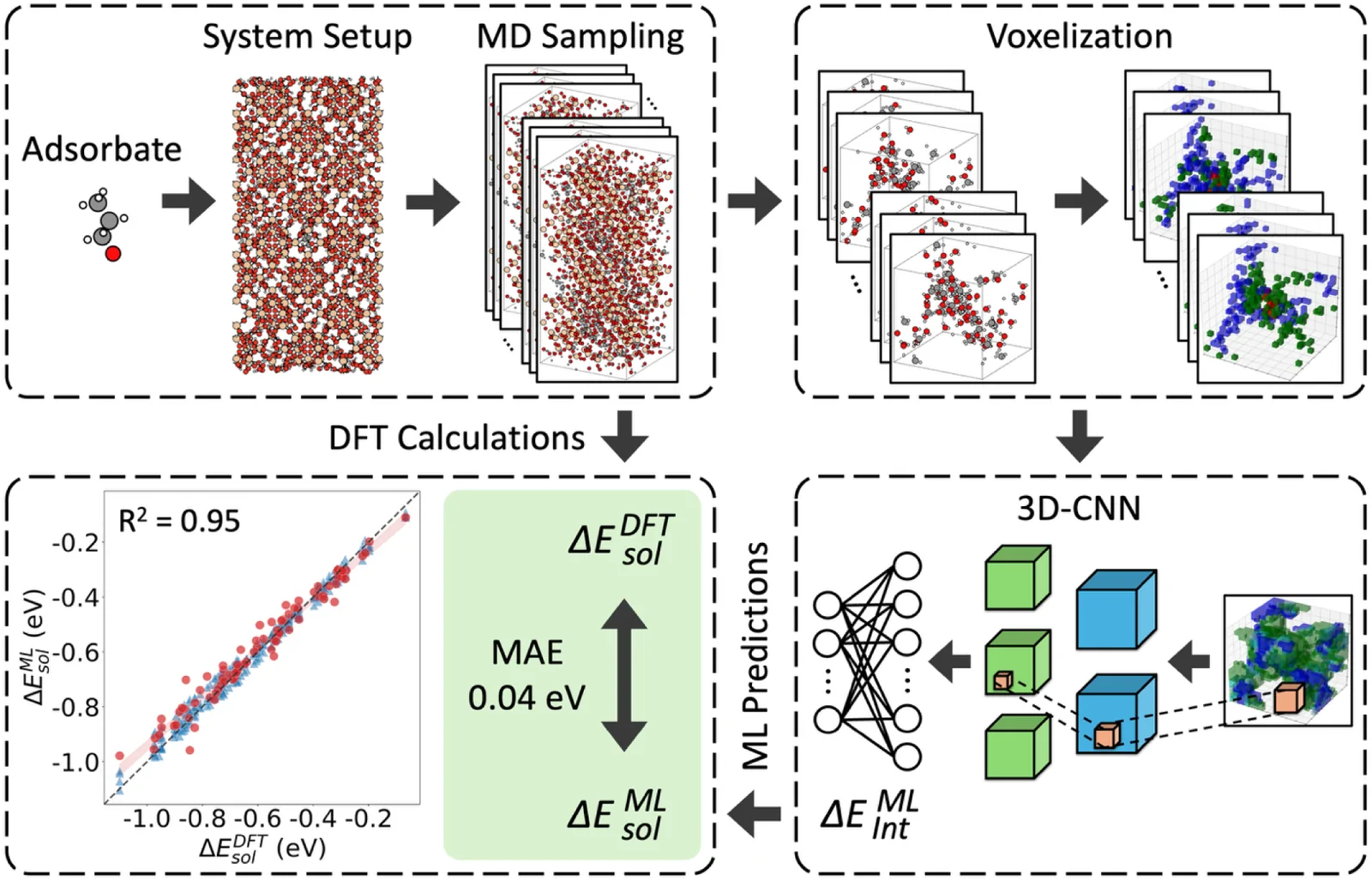
Overview of the workflow employed in this work. System snapshots sampled from classical MD simulations are converted into voxel grid representations, which serve as inputs to a 3D convolutional neural network that predicts adsorbate–solvent interaction energies using DFT-calculated interaction energies as reference labels.

### 2.1 Data Set Preparation

The data set comprises 12 C_1_–C_3_ oxygenates, including methanol, ethanol, propanol, isopropanol, formaldehyde, acetaldehyde, propionaldehyde, ethylene glycol, propylene glycol, 1,3-propanediol, glycerol, and one glycerol intermediate with stoichiometry C_3_H_7_O_3_. A complete inventory of all 80 simulation systems, including their adsorbate/configuration identifiers, pore models, and solvent compositions, is provided in Supporting Information Table S1. Each adsorbate is represented by one or more adsorption configurations in hydrophilic and hydrophobic pore models of Ti-FAU in pure water. FAU is selected because it contains only one symmetrically distinct tetrahedral (T) site, which simplifies the models.^32^ The periodic Ti-FAU unit cell used in this study contains 194 framework Si atoms and one framework Ti atom, corresponding to a Si/Ti ratio of 194. The model is constructed by replacing one framework Si atom at a T site with Ti. Because only one framework Ti atom is present in each periodic unit cell, no neighboring Ti pairs are represented, and the Ti adsorption site is spatially isolated. The same framework composition and Ti-site arrangement are used for all simulation systems. All adsorbates are bound to the Ti site via one of their oxygen atoms. The hydrophilic Ti-FAU pore model contains hydroxyl groups proximal to the Ti site that introduce local polarity.^32^ In the hydrophobic Ti-FAU pore model, the hydroxyl groups are placed in a different pore than the Ti site, maintaining a hydrophobic local environment around the active site but comprising the same overall number of hydroxyl groups as in the hydrophilic pore model. A subset of six adsorbates—methanol, propanol, glycerol, propionaldehyde, ethylene glycol, and propylene glycol—is additionally modeled in water–methanol mixtures with methanol mole fractions of 10%, 20%, and 50%. The mixed-solvent subset focuses on representative C_2_–C_3_ oxygenates spanning different functional groups, with methanol retained as a C_1_ reference species. Thus, the data set does not comprise a full-factorial combination of all adsorbates and solvent compositions. For polyols represented by multiple adsorption configurations in pure water, only the thermodynamically most stable configuration is retained in the mixed-solvent systems.

We define a simulation system as a unique combination of adsorbate identity and adsorption configuration, zeolite pore type, and solvent composition. Solvent configurations for each simulation system are generated in MD with LAMMPS.^33^ The data set contains 44 pure-water systems and 12 systems at each of the three methanol–water compositions (the four solvent-composition groups are unequal in size), yielding 80 simulation systems in total, with 40 systems in each pore type (see Table S1). $\Delta E_{int}^{DFT}$ values are calculated using DFT for a subset of 10 configurations per system, resulting in a total of 800 $\Delta E_{int}^{DFT}$ values.

To improve orientational robustness and expand the training set with symmetry-equivalent inputs, each snapshot is transformed using the 24 proper rotational symmetries of a cube, expanding the 800 DFT-labeled snapshots to 19,200 voxel representations. All 19,200 augmented representations are used during model training, while each set of 24 rotational variants retains the same DFT label. Rotational consistency is evaluated separately through test-time augmentation (TTA) analysis. Details of the augmentation procedure and rotational consistency analysis are provided in Supporting Information, Sections S2.3 and S5.3, respectively. These analyses indicate that the model is robust with respect to rotations, with minimal prediction variance across rotated variants and nearly identical MAE values with and without rotational averaging at inference time.

Values of $\Delta E_{int}^{DFT}$ are calculated following the methods in our prior work,^32^ with further details provided below in Section 2.6. For each simulation system, the 10 values of $\Delta E_{int}^{DFT}$ are averaged to obtain the ensemble-averaged solvation interaction energy, $\Delta E_{sol}^{DFT}$ , yielding 80 ensemble-averaged values in total (see Section 2.6). These values represent the ensemble averages for the system, whereas $\Delta E_{int}^{DFT}$ are the solvation interaction energies for each snapshot. The distributions of $\Delta E_{int}^{DFT}$ and $\Delta E_{sol}^{DFT}$ values across the full data set are shown in Figure 2. Figure 2a shows $\Delta E_{int}^{DFT}$ values ranging from −1.48 eV to +0.06 eV, while Figure 2b shows $\Delta E_{sol}^{DFT}$ values ranging from −1.10 eV to −0.06 eV. Both distributions are approximately unimodal. Figure 2c illustrates the distribution of individual $\Delta E_{int}^{DFT}$ values versus their corresponding ensemble-averaged $\Delta E_{sol}^{DFT}$ values. Because $\Delta E_{sol}^{DFT}$ is the mean of the 10 $\Delta E_{int}^{DFT}$ values for each system, the points are centered around the parity line by construction. Their vertical dispersion reflects configuration-dependent variation in adsorbate–solvent interactions. Although the magnitude of this dispersion varies among systems, no values are clearly isolated, indicating that there are no extreme outliers.

**Figure 2:**
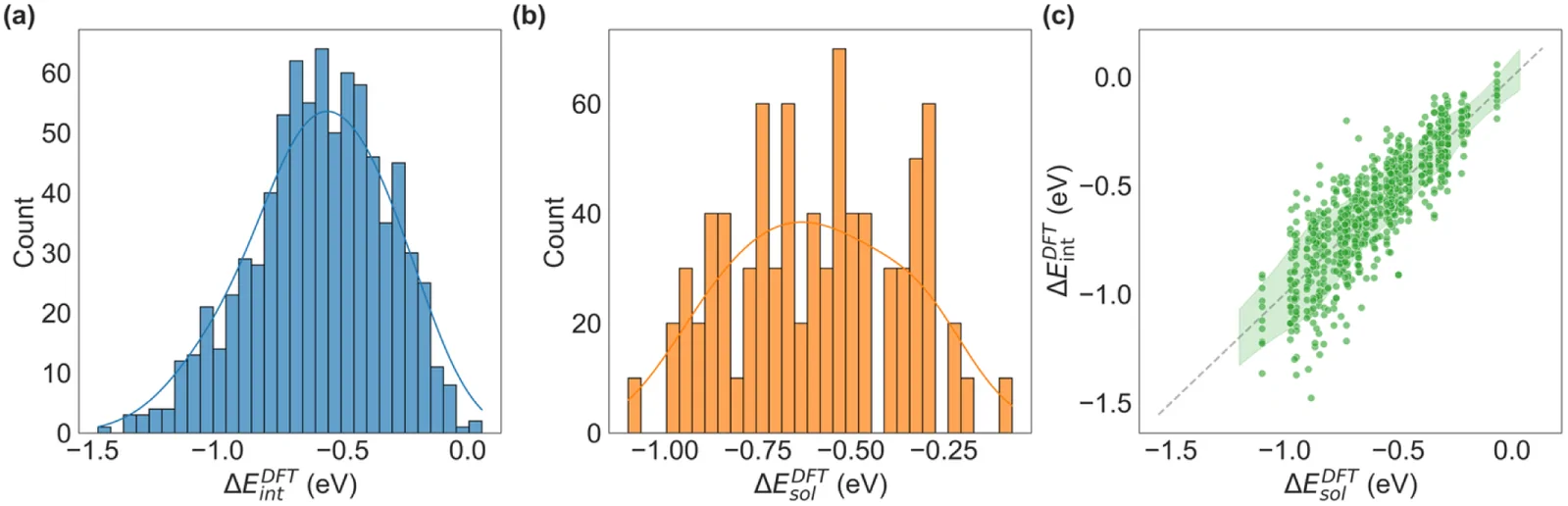
Distributions of $\Delta E_{int}^{DFT}$ (a) and $\Delta E_{sol}^{DFT}$ (b) and scatter plot of individual $\Delta E_{int}^{DFT}$ values against their corresponding $\Delta E_{sol}^{DFT}$ values (c). In (c), the black dashed line is the parity line, and the green shaded region represents ±1 standard deviation in $\Delta E_{int}^{DFT}$ .

### 2.2 Voxel Grid Representations

To prepare input data for deep learning models, the raw MD snapshots are transformed into three-dimensional voxel grids. This approach encodes the atomic coordinates and associated atomic properties from each MD snapshot as a fixed-size, multichannel tensor that serves as input to the 3D-CNN model. Unlike conventional descriptor-based approaches, which rely on chemical intuition and substantial feature engineering, voxelization preserves spatially resolved atomic information and enables the 3D-CNN to learn features for predicting values of $\Delta E_{int}^{DFT}$ automatically. This representation is particularly useful for confined solvent environments such as zeolite pores, which exhibit complex spatial heterogeneity, including variations in solvent density and confinement-induced organization associated with pore geometry, that can be difficult to describe using hand-crafted descriptors.^11^

The workflow for converting MD snapshots into multichannel voxel grid representations is illustrated in Figure 3. It begins with parsing the LAMMPS data and dump files using MDAnalysis.^34,35^ Atoms belonging to different molecular species are then identified from the LAMMPS topology file and assigned to adsorbate, water, and cosolvent (here, methanol) groups. Only the adsorbate and solvent molecules are included in the voxel representation. Zeolite framework atoms, including the framework Ti atom and framework hydroxyl groups, are excluded to focus the input representation on the adsorbate and local solvent configurations and to avoid repeatedly encoding framework atoms that remain fixed within each respective pore model across the MD snapshots.

**Figure 3:**
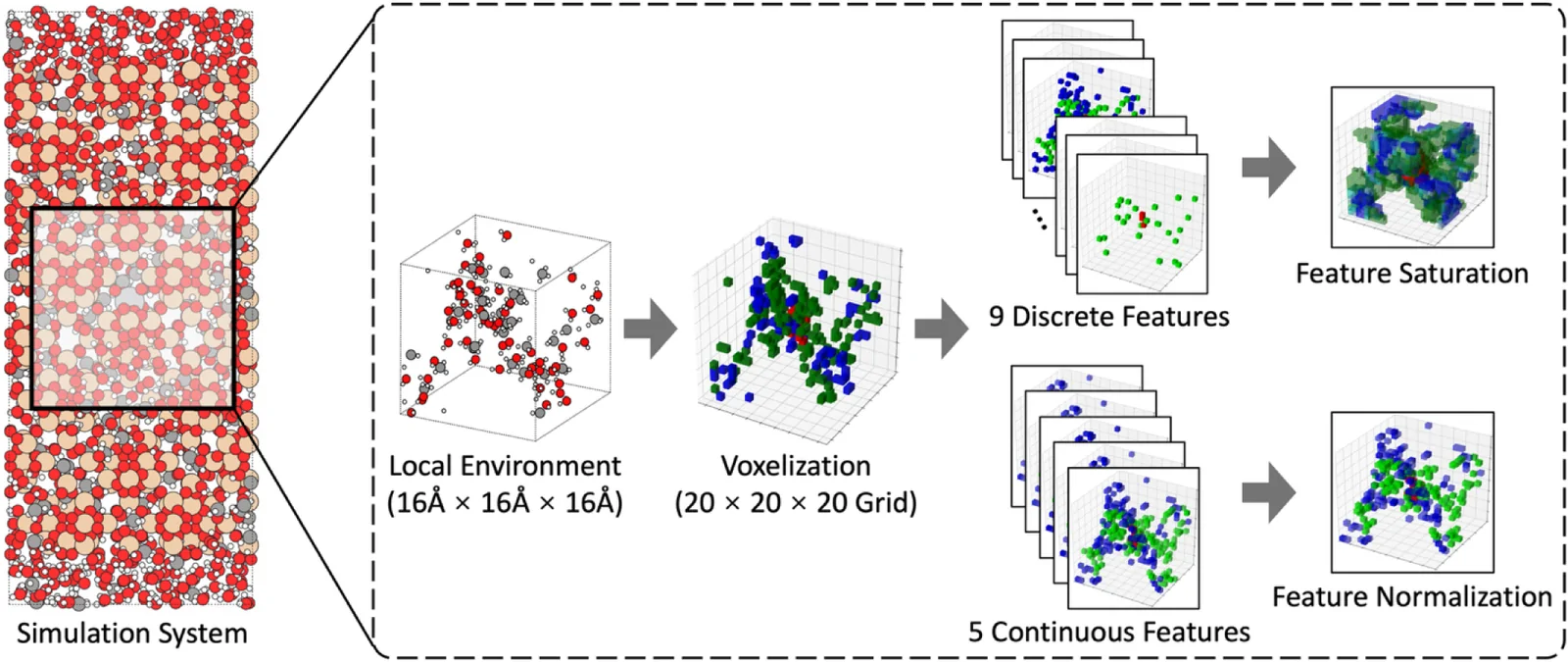
Schematic of the voxelization and feature processing workflow used in this work. Color key: Atoms: O = red, C = gray, H = white, Si = tan, Ti = light gray. Voxels: Adsorbate = red, water = blue, methanol = green.

All systems in the present data set contain Ti as the sole framework heteroatom and have the same framework elemental composition. However, the hydrophilic and hydrophobic pore models differ in the spatial placement of the four framework hydroxyl groups relative to the Ti site. Because these framework features are not explicitly encoded, their effects are represented only indirectly through the resulting adsorbate geometries and solvent configurations sampled within each pore environment. This treatment aligns with prior studies on similar confined systems^11,32,36^ which emphasize the importance of local adsorbate and solvent organization in determining interfacial behavior. Thus, the current input representation does not explicitly encode the number and spatial arrangement of framework hydroxyl groups, or the elemental identity and number of framework heteroatoms.

To construct the voxel-based representations, the atomic coordinates in each MD snapshot are expressed relative to the adsorbate center of mass (COM). This procedure defines an adsorbate-centered cubic box with a side length of 16 Å. For direct coordinate-to-voxel assignment, atoms lying outside the cubic boundaries are excluded, and the retained atoms are mapped to voxels according to their coordinates relative to the adsorbate COM via a 3D histogram operation. We find that increasing the box size beyond 16 Å provides only marginal improvements in accuracy while substantially increasing the input size and training cost. Physically, the chosen 16 Å box side length corresponds to retaining solvent molecules within 8 Å (i.e., the box half-width) of the adsorbate COM. Although solvent structuring beyond this vicinity can influence solvation thermodynamics,^9,37^ values of Δ*E_int_* are expected to be dominated by short- to medium-range interactions.^9^ The choice to use a cutoff of 8 Å is supported by the truncation scheme validated for the DFT calculations, where Chen et al.^32^ demonstrated that interaction energies converge when retaining solvent molecules within a 7 Å radius. The 16 Å cube is discretized into a 20 × 20 × 20 voxel grid, yielding voxels with side lengths of 0.8 Å. Grid-size and resolution tests show that using 1.0 Å voxels with a 20 × 20 × 20 grid, which increases the box side length to 20 Å, does not improve accuracy. Conversely, using 0.5 Å voxels with the same grid reduces the box side length to 10 Å and increases the error, consistent with insufficient spatial coverage. Increasing the grid to 32 × 32 × 32 at a resolution of 0.5 Å restores the 16 Å side length but increases the training time by ∼4× and still yields a higher MAE.

Building on this occupancy grid, the 3D spatial structure is extended into a multichannel tensor, where atomic features are encoded into distinct feature channels. The atomic features are selected to reflect the dominant interactions governing adsorbate–solvent behavior. Fourteen atomic features are employed (Table 1). These features are categorized into two groups: nine discrete features encoded as binary or one-hot indicators and five continuous scalar properties. Detailed definitions and calculation methods for these features are provided in Supporting Information Section S2. The summary is as follows. For the discrete features, elemental identities (C, H, and O) are represented using one-hot encoding, depending only on which element (if any) is present. The physicochemical properties (i.e., hydrophobic character and hydrogen-bond donor/acceptor capabilities) are encoded as binary indicators. Their values depend on which element (if any) is present as well as its covalent connectivity. Specifically, an adsorbate C atom is classified as hydrophobic if it has no directly bonded O neighbor, whereas an adsorbate H atom is classified as hydrophobic if it is bonded to a hydrophobic C atom. Solvent atoms are not assigned hydrophobic character. Hydrogen bond acceptor capabilities are assigned to all O atoms, while hydrogen bond donor capabilities are assigned to H atoms that are covalently bonded to a single O atom neighbor. Three additional configuration-dependent features describe the instantaneous hydrogen-bonding state in each MD snapshot. The general hydrogen-bond indicator is assigned to the donor heavy atom, donor H atom, and acceptor atom participating in a detected hydrogen bond, whereas the donor- and acceptor-specific indicators are assigned to the donor heavy atom and acceptor atom, respectively. Adsorbate–solvent hydrogen bonds in both donor–acceptor directions are identified using MDAnalysis^34,35^ (see Supporting Information Section S2.2 for details). The five continuous features comprise atomic mass, partial charge, Lennard-Jones parameters (ε and σ), and valence number. These features are automatically obtained from or derived from the LAMMPS input (data) and trajectory (dump) files using molecular connectivity and instantaneous atomic coordinates.

**Table 1:** Summary of Atomic Features Used for Voxel Grid Representations

Type	Feature name	Description
Discrete	Atom type C	One-hot encoding for carbon atoms
	Atom type H	One-hot encoding for hydrogen atoms
	Atom type O	One-hot encoding for oxygen atoms
	Hydrophobic atom	Binary indicator for hydrophobic character
	H-bond donor	Binary indicator for hydrogen bond donor capability
	H-bond acceptor	Binary indicator for hydrogen bond acceptor capability
	Is H-bonded	Configuration-dependent binary indicator for participating in a hydrogen bond
	Is H-bonded donor	Configuration-dependent binary indicator for acting as a hydrogen bond donor
	Is H-bonded acceptor	Configuration-dependent binary indicator for acting as a hydrogen bond acceptor
Continuous	Atom mass	Atomic mass in atomic mass units (amu)
	Partial charge	Partial atomic charge (e) used in MD
	Valence	Atomic coordination number based on covalent connectivity
	LJ sigma	Lennard-Jones σ (Å) from MD force field
	LJ epsilon	Lennard-Jones ε (kcal/mol) from MD force field

To explicitly distinguish contributions from adsorbate and solvent atoms, a channel-separation scheme is adopted. This scheme expands the input tensor to 28 channels. The first 14 channels receive contributions exclusively from adsorbate atoms, whereas solvent atoms make no contribution to these channels. Conversely, the remaining 14 channels receive contributions exclusively from solvent atoms, with no contribution from the adsorbate. This separation preserves molecular identity and allows the 3D-CNN to process the adsorbate and solvent representations through separate network branches before combining their learned features. However, channel separation further increases the sparsity inherent in atom-centered voxel representations because atomic features are initially assigned only to voxels containing atomic nuclei and each channel group represents only one component of the system. Consequently, most voxel values are zero, particularly in the adsorbate channel group, where the adsorbate is localized near the grid center. Such highly localized signals provide limited spatial support for the convolutional filters in the early network layers. Moreover, mapping atomic coordinates to discrete voxels introduces discretization sensitivity: a small atomic displacement across a voxel boundary can abruptly move a feature between neighboring voxels. To reduce sparsity and obtain a smoother representation that incorporates the characteristic spatial extent of each atom, the discrete atomic features are spatially broadened using a saturation function parametrized by the atomic van der Waals radius:^38,39^

1 $$n\left(r\right)=1-\exp\left(-{\left(\frac{r_{vdW}}{r}\right)}^{12}\right)$$

where *r_vdW_* is the van der Waals radius of the atom and *r* is the distance from the atom’s nucleus to the voxel center. This saturation function ensures that each atom has maximum influence at its center (n → 1 as r → 0), which rapidly diminishes at distances beyond its van der Waals radius. In this implementation, voxels can contain contributions from multiple atoms. Only the maximum contribution from all contributing atoms is retained for each voxel to prevent atomic overlap. Unlike the discrete features, continuous atomic features (atomic mass, partial charge, valence, and Lennard–Jones parameters) exhibit a range of values and varying scales. To ensure consistent magnitudes across channels, they are therefore normalized independently for each snapshot and feature channel using max-abs scaling:

2 $$x_{normalized}=\frac{x}{\max\left(\left|x\right|\right)}$$

where *x* represents the original feature values in occupied voxels, and max­(|*x*|) is the maximum absolute value across all occupied voxels for that specific feature channel. Max-abs scaling is applied exclusively to occupied voxels (i.e., those containing at least one atom) and results in values in the range [−1, 1]. Empty voxels remain equal to zero.

### 2.3 Neural Networks

The 3D-CNN models are developed and implemented using PyTorch 2.6.0.^40^ Detailed model parameters and specifications are provided in the Supporting Information Section S3. A schematic overview of the 3D-CNN architecture is shown in Figure 4, which illustrates the sequential workflow through the model’s four primary stages: branch-specific feature extraction for the adsorbate and solvent, attention-guided fusion and integration of the extracted features, hierarchical processing of the fused spatial features within a residual CNN backbone, and regression through fully connected layers. Given a voxel grid derived from an MD snapshot, the model produces a scalar prediction of Δ*E_int_*. We note that the present 3D-CNN is a discriminative energy-prediction model rather than a generative model. Given a voxelized adsorbate-solvent configuration, it learns a direct mapping to the corresponding value of Δ*E_int_* and does not generate molecular structures, solvent configurations, or new voxel representations.

**Figure 4:**
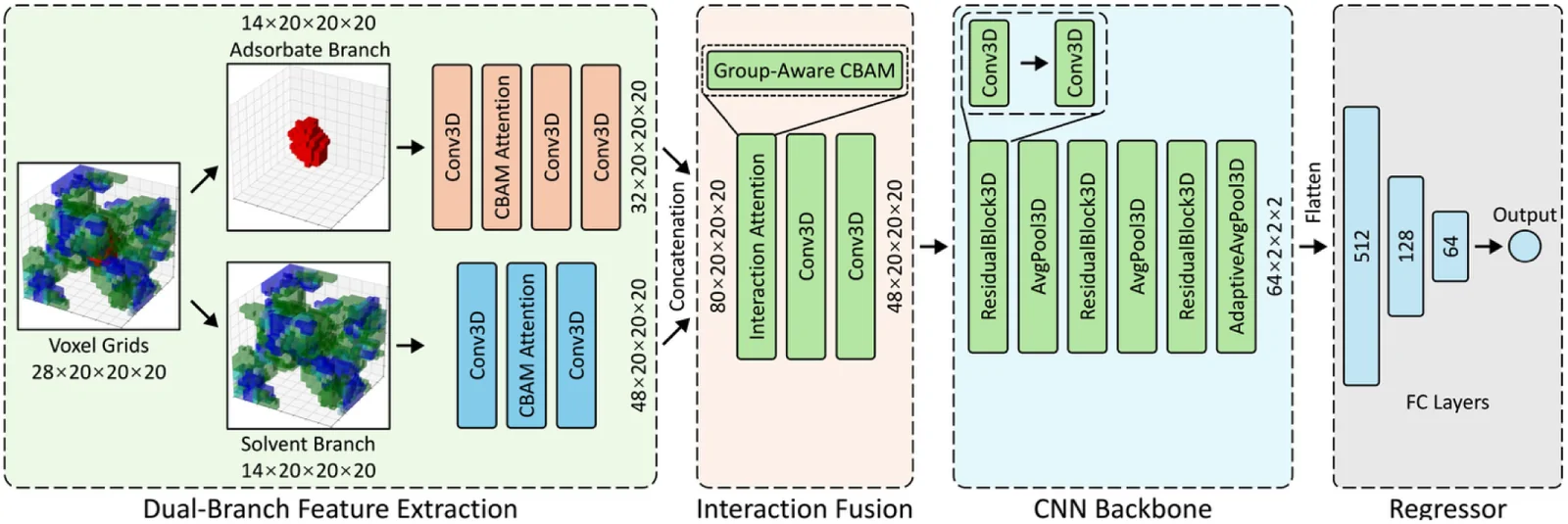
Schematic overview of the 3D-CNN architecture for predicting Δ*E_int_* (output).

The adsorbate and solvent feature channels are first processed through separate branches. This dual-branch architecture enables the model to learn branch-specific spatial representations before combining information from the two molecular components. To adaptively emphasize informative features and spatial regions, a 3D adaptation of the convolutional block attention module (CBAM), originally proposed by Woo et al.^41^ for 2D convolutions, is embedded within each branch. These components are referred to as the adsorbate attention module and the solvent attention module, respectively. Each CBAM sequentially applies channel attention followed by spatial attention, producing input-dependent weights that emphasize informative feature channels and spatial regions while suppressing less relevant signals. Feature extraction in each branch begins with an initial convolutional layer. The design of the kernel sizes for these convolutional layers is guided by the spatial scales of the chemical features in each branch. For the adsorbate branch, the objective is to resolve the sparse, centrally localized features of the adsorbate. Accordingly, it initially employs a 3 × 3 × 3 voxel kernel to capture the local molecular geometry. In contrast, the solvent branch handles more spatially distributed features. It hence begins with a larger 5 × 5 × 5 voxel kernel, allowing the model to capture spatial correlations on the scale of hydrogen-bonding distances. CBAMs are then employed to refine the intermediate features in each branch. In the adsorbate branch, this is followed by a 1 × 1 × 1 voxel kernel that learns cross-channel feature combinations and enriches the otherwise sparse voxel grid, and finally by another 3 × 3 × 3 voxel kernel to integrate spatial information. In the solvent branch, the CBAM is followed by a 3 × 3 × 3 voxel kernel to integrate and refine the extracted features. Across both branches, each convolutional layer is followed by batch normalization to stabilize training, and a rectified linear unit (ReLU) activation function to introduce nonlinearity.

Following independent feature extraction, the feature maps generated by the adsorbate and solvent branches are concatenated (fused) along the channel dimension to form a single tensor that combines adsorbate-derived and solvent-derived channel groups. The tensor is passed to the interaction attention module, which is a third attention module specifically designed to emphasize adsorbate–solvent interactions through a group-aware attention mechanism. Separate subnetworks process the adsorbate-derived and solvent-derived channel groups independently, while learnable interaction-strength parameters modulate how strongly the attention of one group is influenced by the other to capture their cross-channel dependencies. This dynamically reweighted channel information subsequently guides the spatial attention mechanism, allowing the network to highlight specific regions where adsorbate structural motifs and local solvent structures jointly correlate with Δ*E_int_*. Following the group attention step, an interaction convolution module integrates the reweighted features using sequential 3 × 3 × 3 voxel and 1 × 1 × 1 voxel kernels. The former captures spatial correlations among the most important features, while the latter performs channel-wise feature recombination to yield a unified tensor, which serves as the input to the main CNN backbone.

The CNN backbone consists of three consecutive residual blocks (adapted from He et al.^42^) that progressively learn hierarchical representations of the adsorbate–solvent interactions. To progressively reduce spatial dimensions while aggregating local features, average pooling layers with a 2 × 2 × 2 kernel and a stride of 2 are applied after the first two residual blocks, halving the spatial resolution after each block. After the final residual block, an adaptive average pooling layer further compresses the remaining spatial dimensions to a spatial size of 2 × 2 × 2. The resulting tensor is then flattened into a 512-dimensional vector and passed to a three-layer fully connected regressor. This regressor consists of two hidden layers with 128 and 64 neurons, respectively, each followed by batch normalization for training stability and ReLU activation, with dropout applied for regularization. The final output layer contains a single neuron that produces the scalar prediction of $\Delta E_{int}^{ML}$ .

Although the initial convolutional layers extract features from local voxel neighborhoods, the model’s final prediction incorporates information from the complete voxelized domain. Successive convolutions and two average-pooling operations progressively enlarge the receptive field, such that the deepest convolutional features have a theoretical receptive field spanning the full 20 × 20 × 20 input grid. In addition, the CBAM channel-attention modules use global average and maximum pooling over the spatial dimensions. The final adaptive pooling operation retains a 2 × 2 × 2 representation of the input domain, and all eight spatial regions are jointly processed by the fully connected regressor. Consequently, the model is not restricted to independently evaluated nearest-neighbor environments, and spatially separated regions within the 16 Å input box can jointly affect the scalar energy prediction. At lower solvent loadings, unoccupied regions would be represented by zero-valued voxels, while spatially separated solvent molecules remaining within the input box would remain explicitly represented.

### 2.4 Training and Evaluation

Data partitioning and the calculation of evaluation metrics are handled using the scikit-learn library.^43^ Model training is performed on NVIDIA A100 GPUs via CUDA 12.4 for accelerated computation. Further, all custom scripts are available at https://github.com/getman-research-group/3dcnn-zeolite-solvation for reference. The models are trained and evaluated using three complementary data-partitioning strategies—the simulation-system split, solvent-composition split, and pore-type split—to evaluate predictive performance under different holdout conditions. In all strategies, the set of voxel grid representations derived from each simulation system is treated as an indivisible unit during data partitioning, guaranteeing that all snapshots (and their augmented variants, where appropriate) from the same system are assigned exclusively to either the training or test set in each fold, preventing data leakage.

The simulation-system split is the primary evaluation strategy and is implemented using 5-fold grouped cross-validation, as illustrated in Figure 5a. The grouping unit is an individual simulation system, identified by the unique combination of solvent composition, pore type, and adsorbate/configuration identifiers. Each of the 80 simulation systems is assigned to exactly one test fold. In each fold, 64 systems are used for training, and the remaining 16 systems are used for testing. All 10 MD snapshots from a given simulation system, together with their rotationally augmented voxel representations, remain in the same fold. Systems containing the same chemical adsorbate may therefore occur in both the training and test sets when they differ in solvent composition, pore type, or adsorption configuration. Consequently, the simulation-system split evaluates generalization to held-out simulation systems within the represented chemical domain, but it does not establish extrapolation to an entirely unseen adsorbate species or chemical family. The solvent-composition and pore-type splits, illustrated in Figure 5b, evaluate predictive performance when an entire solvent composition or pore type is excluded from the corresponding training set. The solvent-based split strategy divides the data set into 4 folds corresponding to distinct solvent environments: pure water, 10% methanol–water mixture, 20% methanol–water mixture, and 50% methanol–water mixture. In each split, the model is trained on data from three solvent compositions and tested on the remaining one, and this procedure is repeated so that each composition serves as the test set once. Similarly, the pore-type split employs a 2-fold CV, training on data from one pore type (e.g., hydrophilic) and testing on the other (e.g., hydrophobic). The solvent and adsorbate compositions and the number of simulation systems in each test fold under all three data-split strategies are summarized in Supporting Information Table S2. For each partitioning strategy, the final performance scores are calculated as the unweighted arithmetic means of the fold-specific evaluation metrics. Consequently, each held-out solvent composition contributes equally to the reported solvent-composition split results, irrespective of the number of systems in that fold. No solvent-composition weighting, oversampling, or downsampling is applied during model training.

**Figure 5:**
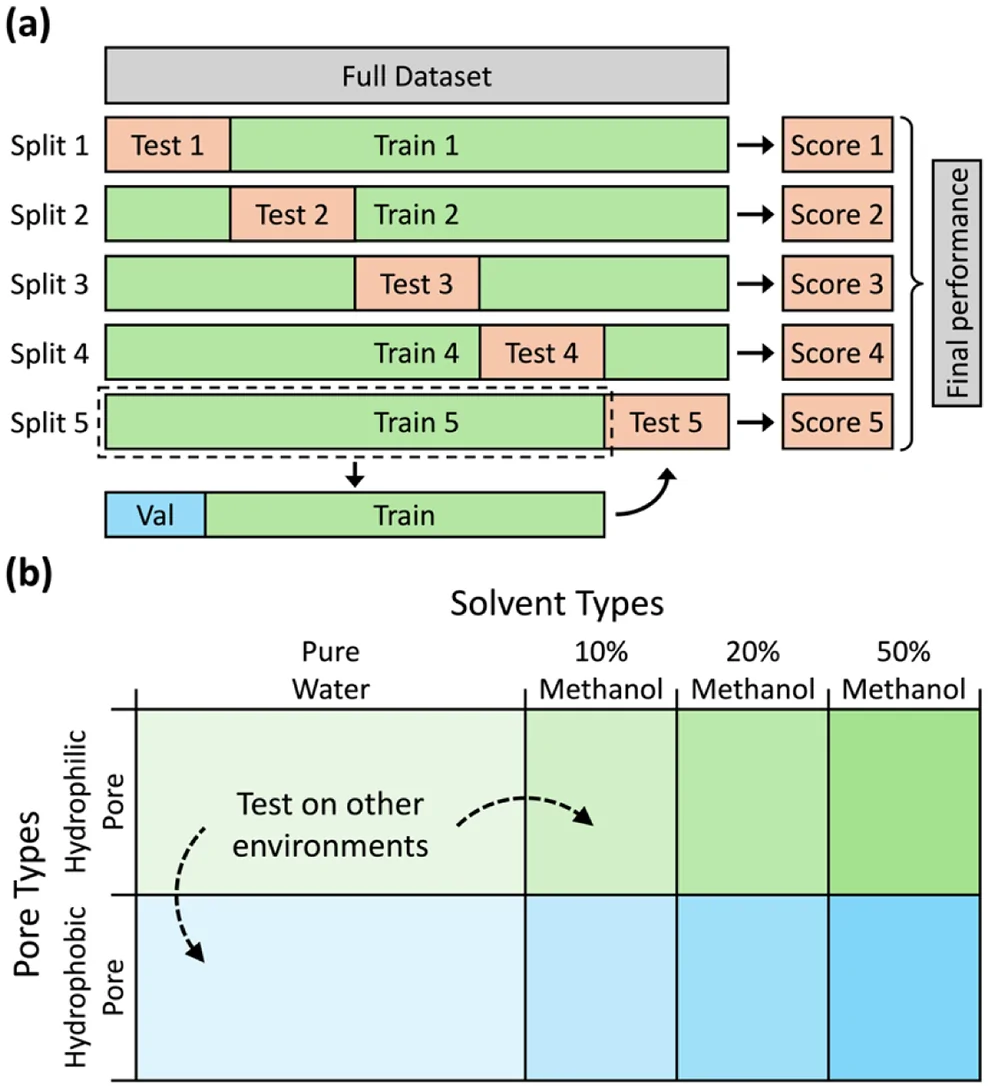
Model training and evaluation strategies: (a) 5-fold simulation-system split and (b) solvent- and pore-type-based cross-validation schemes.

Each CV strategy follows a standardized training protocol. Within each fold, the target values $\Delta E_{int}^{DFT}$ are normalized using a standard scaler fitted exclusively to the training set to prevent data leakage from the test set. The fitted scaler is then used to transform the test-set labels, ensuring that the training and test data are on the same numerical scale. The model is trained for 200 epochs with a batch size of 32, using the AdamW optimizer,^44^ which decouples weight decay from the gradient updates, and uses the mean squared error (MSE) as the loss function. A learning rate of 2.0 × 10^–4^ is used, along with a weight decay of 1.0 × 10^–4^ for regularization. Gradient clipping with a maximum norm of 0.5 is applied to maintain training stability. During evaluation, the model’s predictions are inverse-transformed back to the original energy scale using the same fitted scaler as used before computing the performance metrics.

Predictive performance is evaluated by comparing the predictions against both $\Delta E_{int}^{DFT}$ and $\Delta E_{sol}^{DFT}$ . Performance is quantified using three standard metrics: coefficient of determination (R^2^), mean absolute error (MAE), and root mean squared error (RMSE). Definitions and explicit expressions for these metrics are provided in Supporting Information Section S4.1. Following our prior work,^45^ an R^2^ value greater than 0.8 is considered indicative of good predictive performance, while an MAE below 0.10 eV is set as the criterion for chemical accuracy, consistent with the precision of the reference MSS method.^46,47^

### 2.5 Data Interpretation

To elucidate how the 3D-CNN prioritizes spatial regions within the voxelized molecular environments, we analyze the spatial attention weights produced by the CBAMs. This analysis is performed independently for the adsorbate branch, the solvent branch, and the adsorbate–solvent interaction module. The attention maps generated within each CBAM represent the learned voxel-wise importance weights applied during the feature reweighting operation. Specifically, the spatial attention map *M_S_* is computed by applying channel-wise average pooling and max pooling to the intermediate feature map $F\in R^{C\times D\times H\times W}$ (where C = number of channels, and D, H, W represent the 20 × 20 × 20 voxel grid). This process compresses the channel dimension into two single-channel 3D maps, which are concatenated along the channel dimension and passed through 3D convolution followed by a sigmoid activation. This yields a single-channel spatial weight map $M_{S}\in R^{1\times D\times H\times W}$ with values in [0, 1]. Because all convolutional layers preceding the CBAMs employ padding that preserves the spatial dimensions, each attention map retains the original 20 × 20 × 20 voxel grid resolution and can be directly mapped onto the physical coordinate system of the input domain. For visualization, the spatial attention maps are extracted via forward hooks during inference on the trained model. To translate these 3D attention tensors into interpretable 2D heatmaps, orthogonal projections along the three principal axes are obtained by averaging the four central voxel layers for each viewing direction. This generates three cross-sectional views through the geometric center of the grid that collectively intersect the adsorbate and surrounding solvent molecules in the central solvation region, providing complementary perspectives that illustrate the core interaction patterns.

In addition to the spatial attention analysis, we employ integrated gradients to quantify the importance of the individual input feature channels. This method computes feature attributions by accumulating gradients along a straight-line path from a zero-valued baseline input to the actual input, effectively measuring how each feature contributes to $\Delta E_{int}^{ML}$ . For each channel, the overall importance score is defined as the spatial mean of its absolute attribution values across the voxel grid. Scores are normalized to the global maximum across all channels to yield relative importance. Together, the spatial attention maps and channel-level attributions provide complementary views of the model’s learned representations: the former reveals where the model focuses within the voxel grid, while the latter identifies which input features drive the prediction.

### 2.6 Details of the MSS Simulations

Values for $\Delta E_{int}^{DFT}$ for adsorbates in pure-water solvent are obtained from Chen et al.,^32^ while those in water–methanol solvent mixtures are newly generated for this work following the same MSS protocols. Pore models and simulation setup used in this work are adopted from Chen et al.^32^ and illustrated in Supporting Information Section S1. The workflow is as follows. The Ti-FAU models are constructed according to Chen et al.^32^ Adsorbate geometries are first optimized using DFT. During this initial setup step, the adsorbate molecules, Ti atoms and adjacent oxygen atoms and hydroxyl groups are relaxed, while the remainder of the zeolite atoms as well as the zeolite framework are held fixed. The zeolite is then cleaved, leaving a vacuum region between periodic images. A total of 1,200 solvent molecules is added to this region, and their positions along with the simulation box volume are equilibrated in MD using the NPT ensemble (constant number of molecules, pressure, and temperature). The temperature is set to 300 K and the pressure to 1 atm in these simulations. Equilibrium is confirmed when the density of the bulk solvent (i.e., the region outside the zeolite framework where layering on the zeolite-water interface has diminished) stabilizes at values consistent with the density of the bulk solvent (i.e., without the zeolite). Production simulations in the NVT ensemble (constant number of molecules, volume, and temperature) are then conducted at 300 K. These consist of a 10 ns equilibration phase followed by a 5 ns production run. During all MD simulations, the zeolite and adsorbate atoms are kept fixed, while solvent molecules are allowed to move. Force fields include TIP3P/CHARMM^48,49^ for water, OPLS-AA^50^ for adsorbates, TraPPE-Zeo^51^ for zeolite Si and O, and a potential derived from TiO_2_ for Ti and adjacent O (see Chen et al.^32^). All MD simulations are conducted using the Large-scale Atomic/Molecular Massively Parallel Simulator (LAMMPS).^52^ The MD-derived solvation energy, $\Delta E_{sol}^{MD}$ , is calculated by taking the ensemble average over 5,000 equally spaced snapshots from the 5 ns NVT production run. $\Delta E_{int}^{DFT}$ are calculated using DFT. Configurations are sampled every 0.5 ns from the production run in the NVT simulations, yielding 10 MD snapshots per system. To ensure computational tractability, the simulation unit cell used in MD, which was created using a 1 × 1 × 2 supercell of the FAU structure, is truncated to a 1 × 1 × 1 unit cell for DFT. Further, solvent molecules outside of a 7 Å radius from the adsorbate COM are removed, leaving 10–25 solvent molecules per system. $\Delta E_{int}^{DFT}$ is then computed as

3 $$\Delta E_{int}^{DFT}=\left(E_{zeo+ads}^{liq}-E_{zeo+ads}^{vac}\right)-\left(E_{zeo}^{liq}-E_{zeo}^{vac}\right)$$

where $E_{zeo+ads}^{liq}$ is the electronic energy of the zeolite structure with the adsorbate in the presence of solvent molecules, $E_{zeo+ads}^{vac}$ is the electronic energy of the zeolite structure with the adsorbate in the absence of solvent molecules, $E_{zeo}^{liq}$ is the electronic energy of the zeolite structure in the presence of solvent molecules but with the adsorbate removed, and $E_{zeo}^{vac}$ is the electronic energy of the zeolite structure with no adsorbate or solvent molecules. To compute $E_{zeo+ads}^{liq}$ , the adsorbate geometry is relaxed while the zeolite framework and solvent molecules are held fixed. The geometries for the remaining three terms are derived directly from this optimized configuration and evaluated via single-point energy calculations: $E_{zeo+ads}^{vac}$ is computed by removing solvent molecules, $E_{zeo}^{liq}$ by removing the adsorbate, and $E_{zeo}^{vac}$ by removing both solvent and adsorbate. The ensemble-averaged DFT solvation interaction energy for a simulation system, $\Delta E_{sol}^{DFT}$ , is calculated by averaging the 10 values of $\Delta E_{int}^{DFT}$ :

4 $$\Delta E_{sol}^{DFT}=\frac{1}{10}\overset{10}{\underset{i=1}{\sum }}\Delta E_{int,i}^{DFT}$$

where *i* denotes an individual MD snapshot, and the value of 10 in the denominator denotes that 10 DFT snapshots were evaluated for each system. The snapshots are sampled at 0.5 ns intervals and assigned equal weights in the arithmetic average.^31^

DFT calculations are performed using CP2K.^53^ Electronic structure is calculated using the PBE exchange-correlation functional with DZVP-MOLOPT-SR-GTH basis sets, GTH pseudopotentials, D3­(BJ) dispersion correction, and a 360 Ry plane-wave cutoff; Brillouin zone sampling is restricted to the Γ-point. Further details are identical to those in Chen et al.^32^

## 3 Results

Parity plots of the prediction results using the simulation-system split are presented in Figure 6. Specifically, Figure 6a shows $\Delta E_{int}^{ML}$ vs $\Delta E_{int}^{DFT}$ and Figure 6b shows $\Delta E_{sol}^{ML}$ vs $\Delta E_{sol}^{DFT}$ . As shown in Figure 6a, the model achieves robust predictive accuracy at the snapshot level, with values for R^2^ of 0.82, MAE of 0.083 eV, and RMSE of 0.11 eV for the test set. The model’s performance is further enhanced through ensemble averaging, as shown in Figure 6b, where R^2^ improves to 0.95, MAE to 0.04 eV, and RMSE to 0.05 eV. This improvement arises because averaging over multiple solvent configurations reduces the stochastic noise inherent in individual configurations. The red-shaded regions in Figure 6a and b represent the 95% confidence intervals of the fitted regression lines for the test data. The narrowness of these intervals indicates that the relationship between predicted and reference values is statistically well-determined across the test set.

**Figure 6:**
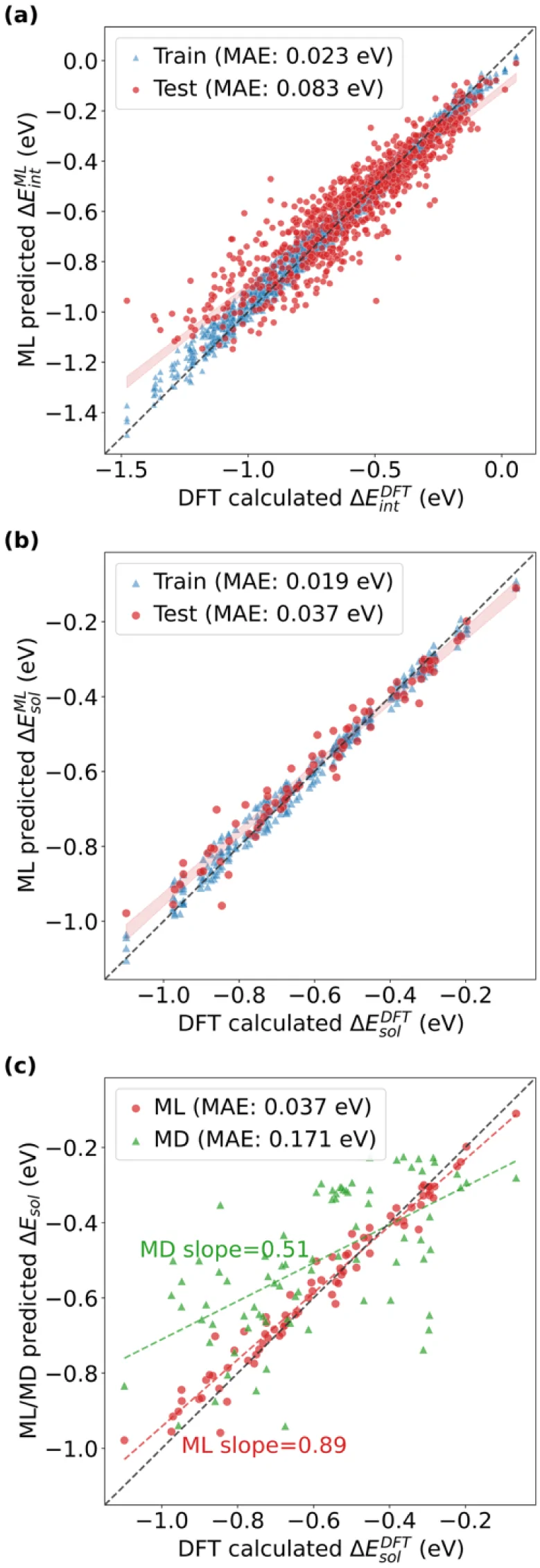
Parity plots of (a) $\Delta E_{int}^{ML}$ vs $\Delta E_{int}^{DFT}$ , (b) $\Delta E_{sol}^{ML}$ vs $\Delta E_{sol}^{DFT}$ , and (c) $\Delta E_{sol}^{ML}$ and $\Delta E_{sol}^{MD}$ vs $\Delta E_{sol}^{DFT}$ . In (a) and (b), the red-shaded area represents the 95% confidence interval for the test set regression.

Figure 6c compares $\Delta E_{sol}^{ML}$ and $\Delta E_{sol}^{MD}$ against the $\Delta E_{sol}^{DFT}$ reference values, where $\Delta E_{sol}^{MD}$ are calculated with the classical force field employed in the MD simulations within the MSS workflow. This comparison is included since it is an efficient way to compute $\Delta E_{sol}$ that can potentially bypass the more computationally demanding DFT calculations. $\Delta E_{sol}^{MD}$ exhibits an MAE of 0.17 eV, which is substantially higher than the 0.04 eV obtained for $\Delta E_{sol}^{ML}$ . Additionally, a linear fit of the predicted values shows a slope of 0.51 for $\Delta E_{sol}^{MD}$ , which is smaller than the slope of 0.89 obtained for $\Delta E_{sol}^{ML}$ . The CNN is trained at the snapshot level: each input is a voxelized molecular configuration sampled using classical force-field MD, and its associated supervised target is the interaction energy obtained using the DFT workflow. The CNN therefore learns a mapping from structural information in the force-field-MD-sampled configurations to the selected DFT energetic target. Collectively, these results show that $\Delta E_{sol}^{ML}$ predictions are more closely aligned with the DFT reference values than $\Delta E_{sol}^{MD}$ . Because agreement with the DFT target is the objective of supervised training, this closer agreement should not be interpreted as evidence that either the CNN predictions or DFT is physically superior to the classical force field. For comparison, in our prior study on adsorbates at Pt(111)/water interfaces,^45^ $\Delta E_{sol}^{MD}$ achieved an MAE of 0.07 eV, which is substantially lower than the 0.17 eV observed here for zeolite pores, suggesting a greater difference between classical and quantum methods in these confined systems.

The model’s predictive performance across the three partitioning strategies is presented in Table 2. The corresponding parity plots for all data-split strategies are provided in Supporting Information Section S5.2. The solvent-based split exhibits performance comparable to the simulation-system split, with test-set MAE values of 0.083 eV for Δ*E_int_* and 0.036 eV for Δ*E_sol_*, indicating useful predictive performance for a solvent composition excluded from the corresponding training fold. These reported values are the unweighted arithmetic averages of the metrics calculated separately for the four held-out solvent compositions; the fold-specific results are provided in Supporting Information Table S6. The pore-type split shows slightly higher errors, with MAE values of 0.106 eV for Δ*E_int_* and 0.059 eV for Δ*E_sol_*. This increase is anticipated, as the 2-fold partitioning restricts the training set to only 50% of the total data and requires the model to generalize between the two pore environments (hydrophilic vs hydrophobic) represented in the data set. This observation aligns well with the notable differences in solvation thermodynamics between hydrophilic and hydrophobic environments reported by Chen et al.^32^ Notably, ensemble averaging over snapshots for Δ*E_sol_* consistently improves overall metrics across all splitting strategies. Overall, these results demonstrate predictive performance for held-out simulation systems, solvent compositions, and pore types within the represented chemical domain; extrapolation to adsorbate species entirely absent from training were not evaluated in this work.

**Table 2:** Model Performance under Three Data-Split Strategies^a^

	Δ*E_int_* (MD snapshot)			Δ*E_sol_* (system-averaged)		
	RMSE (eV)	MAE (eV)	R^2^	RMSE (eV)	MAE (eV)	R^2^
Simulation-System Split	0.110	0.083	0.821	0.047	0.037	0.951
Solvent Split	0.110	0.083	0.802	0.048	0.036	0.942
Pore Type Split	0.133	0.106	0.732	0.071	0.059	0.891

a The simulation-system split uses five-fold grouped cross-validation, with all snapshots from each system retained in the same fold; the solvent-composition split holds out each of the four solvent compositions in turn; and the pore-type split holds out each of the two pore environments in turn. Reported values are the unweighted arithmetic means of the fold-specific metrics.

## 4 Discussion

Beyond achieving low prediction errors, a critical objective of this work is to elucidate how the 3D-CNN model extracts features from the voxelized adsorbate–solvent environments, as this provides insight into the phenomena that drive solvent effects in confined systems. Accordingly, we explore both spatial attention maps and feature channel attributions. Spatial attention weights are analyzed to identify which spatial regions within the local environment are prioritized by the network when predicting Δ*E_int_*. This approach thereby provides a means to identify influential solvent regions, potentially informing the regions to be treated quantum-mechanically in multiscale simulations. The spatial attention weights for a representative system—propylene glycol in the hydrophilic pore model with pure water solvent—are shown in Figure 7. Additional spatial importance analyses for this system based on integrated gradient attributions are provided in Supporting Information Section S5.4. Propylene glycol in pure water solvent is chosen because it exhibits an intermediate interaction energy and a clearly resolved solvent distribution. The attention maps for this system reveal pronounced spatial selectivity patterns, with consistently higher weights in adsorbate-proximal regions and lower weights in peripheral areas. In the adsorbate branch, the attention mechanism concentrates on the volume occupied by the adsorbate and its immediate vicinity. In contrast, the solvent branch exhibits a complementary pattern, with attention focused primarily on the first solvation shell surrounding the adsorbate, where adsorbate–solvent interactions are most pronounced. Overlaying atomic positions on the attention heatmaps reveals that peripheral solvent molecules receive substantially lower attention weights than those in the first solvation shell, despite occupying voxels within the same grid. Further, a slight spatial broadening of attention weights beyond the regions directly occupied by the displayed atoms is observed, arising from the van der Waals-based spatial broadening of discrete atomic features during voxelization, local feature aggregation by the convolutional and spatial-attention kernels, and smoothing in the two-dimensional visualization. This overall spatial pattern is consistent with the known distance dependence of adsorbate–solvent interactions, suggesting that the model has learned to prioritize regions where direct molecular contacts govern solvation energetics. Such spatial selectivity may also inform quantum-chemical modeling efforts by highlighting the solvent regions most influential to the predicted energetics. A comparison of the attention magnitude scales in Figure 7 reveals that the interaction attention module exhibits a broader range and strengthened attention values compared to the solvent attention module. This expanded range indicates enhanced spatial discrimination arising from the joint processing of adsorbate and solvent feature representations.

**Figure 7:**
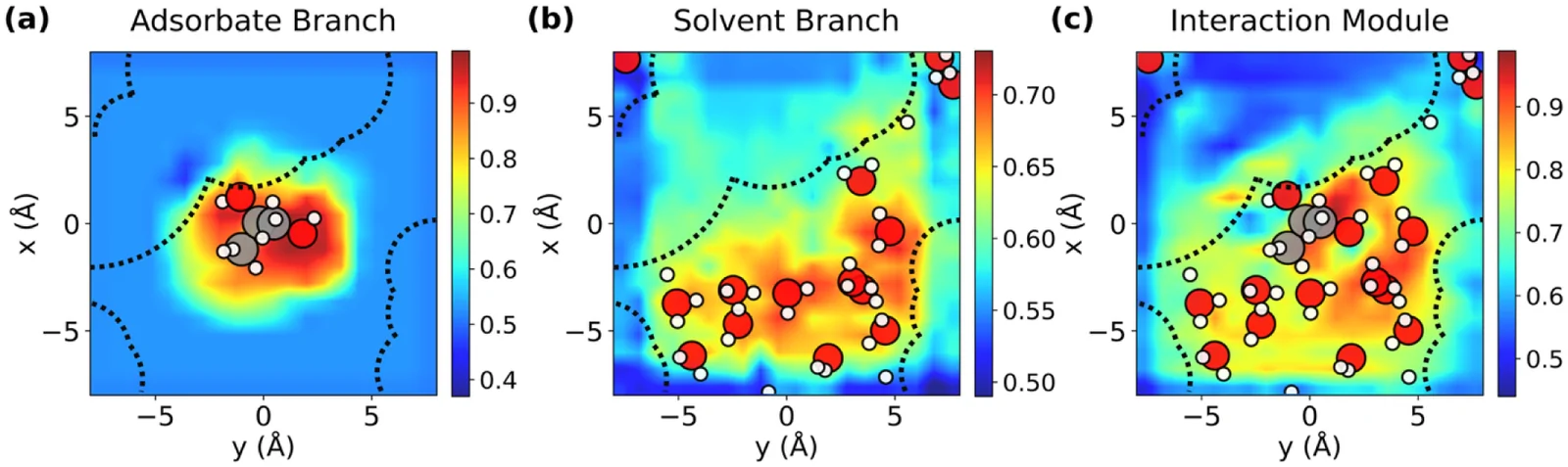
Spatial attention heatmaps for propylene glycol in the hydrophilic pore with pure water solvent: (a) adsorbate, (b) solvent, and (c) interaction attention heatmaps. Black dashed contours mark the zeolite pore boundaries derived from framework atoms within the same slice region.

In addition to the spatial attention maps, we further quantify the relative contribution of different input feature channels using integrated gradients. Figure 8 shows the channel importance analysis results derived from integrated gradients applied to the input voxel features. The analysis is conducted on a representative system, propylene glycol adsorbed in a hydrophilic zeolite pore under a methanol–water mixed-solvent environment, chosen because propylene glycol provides both hydrogen-bond donor and acceptor sites and contains hydrophobic carbon atoms, while the mixed solvent environment includes all possible solvent atom-type channels, thereby allowing a comprehensive evaluation of importance across all atomic feature channels. The results reveal a clear hierarchy of feature importance. Discrete features show substantially higher importance scores than continuous features, with atomic identity features (one-hot encodings of C, H, and O) dominating both the adsorbate and solvent channels. This pattern reflects the fundamental role of atom-type information in defining molecular structure and spatial occupancy. Hydrogen-bond-related binary features also exhibit substantial importance, despite originating from only a small subset of atoms involved in hydrogen-bonding interactions. This indicates that the model has learned to identify these spatially localized but energetically critical interactions. This agrees with findings in Chen et al.,^32^ where $\Delta E_{int}^{DFT}$ was found to correlate well with the number of adsorbate-solvent hydrogen bonds. In contrast, continuous features contribute lower importance magnitudes. This distinction arises as a direct consequence of the spatial transformation strategy applied during voxel grid generation. Specifically, discrete binary features are spatially broadened using a continuous decay function based on van der Waals radii (eq 1), causing a single atom to activate a cluster of neighboring voxels. Conversely, continuous features are mapped strictly to the voxel containing the atomic center, remaining spatially localized. Since the channel importance score is calculated by averaging absolute attributions across the entire spatial grid, discrete features, which possess a significantly larger spatial footprint, yield higher spatially averaged magnitudes than spatially localized continuous features.

**Figure 8:**
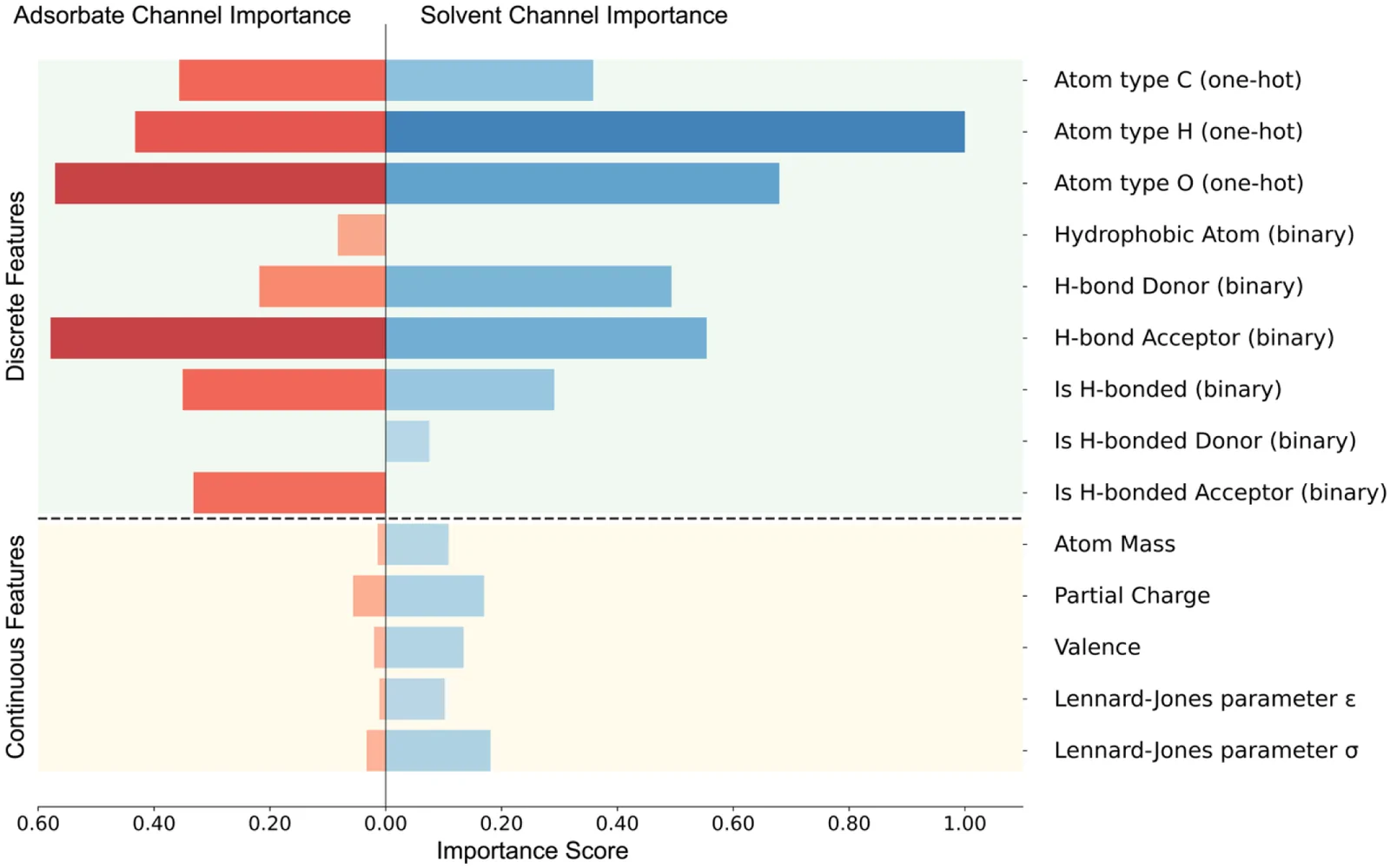
Normalized importance scores for adsorbate (left) and solvent (right) channels derived from integrated gradients attributions for propylene glycol adsorbed in a hydrophilic zeolite pore under a methanol–water mixed-solvent environment.

One possible interpretation of the lower importance scores of the continuous features is that they may be redundant with information already present in the discrete features. For example, the elemental identities and spatial arrangements encoded by the one-hot atom-type features are sufficient to distinguish water molecules from methanol molecules within the mixed-solvent systems considered here. This raises the question of whether the continuous features provide information beyond that already encoded by the discrete features. This question is particularly relevant for the solvent channels because the continuous features exhibit their largest attribution magnitudes in the solvent branch (Figure 8), whereas the corresponding adsorbate-channel attributions are comparatively small. A natural interpretation is therefore that the solvent-side continuous features may primarily function as identifiers of water and methanol molecules rather than contributing additional information relevant to $\Delta E_{int}^{DFT}$ . Importantly, because these attribution magnitudes are influenced by the voxelization scheme and spatial averaging procedure, the lower spatially averaged attribution values of the continuous channels should not by themselves be interpreted as evidence that these features are redundant or can be removed without affecting predictive accuracy. Furthermore, attribution methods such as integrated gradients do not necessarily assign all importance to a single feature when multiple input channels contain related information. Instead, attribution can be distributed among correlated features, causing the apparent importance of any individual channel to be lower than its actual contribution to model performance. For example, although atomic mass is closely related to elemental identity in the present C/H/O data set, partial charge, Lennard-Jones parameters, and valence encode information that is not explicitly represented by the one-hot channels. Similarly, a water oxygen atom and a methanol oxygen atom share the same oxygen identity channel, while differences in their local chemical environments must be inferred from surrounding spatial patterns. Partial charges and Lennard-Jones parameters directly encode distinctions associated with the underlying force-field atom types, while valence provides information about local bonding environments. Thus, although the discrete atom-type and spatial features are sufficient to distinguish water from methanol, the continuous solvent features provide complementary physicochemical information rather than functioning solely as alternative solvent identifiers. Taken together, these considerations indicate that channel-importance scores alone are insufficient to determine feature necessity. Figure 8 should therefore be interpreted as describing how the trained model utilizes the available input channels rather than as a direct test of whether any particular feature could be removed without affecting model performance.

Finally, the predicted quantities should be interpreted within the scope of the MSS workflow. The 3D-CNN directly predicts snapshot-level DFT adsorbate–solvent interaction energies, while Δ*E_sol_* is the arithmetic mean of the interaction energies for 10 sampled configurations of a simulation system. These quantities do not constitute complete solvation free energies and do not explicitly include entropic or other contributions required for a full free-energy evaluation; accordingly, the model accelerates the DFT interaction-energy component of MSS rather than replacing the complete thermodynamic workflow.

## 5 Conclusions

In this study, we developed a machine learning framework that integrates voxel grid representations with an attention-enhanced 3D-CNN model to predict Δ*E_int_* for C_1_–C_3_ oxygenates in hydrophilic and hydrophobic pores of a Ti-FAU zeolite model under aqueous and water–methanol mixed-solvent conditions. By converting MD snapshots into multichannel voxel grids, the framework automates feature extraction and enables the model to learn directly from atomistic configurations. The 3D-CNN reproduces DFT-calculated adsorbate-solvent interaction energies with MAEs of 0.08 eV for individual configurations and 0.04 eV for ensemble-averaged solvation interaction energies. The model therefore provides an efficient surrogate for the DFT interaction-energy calculations within the MSS workflow, substantially reducing their computational cost.

These results validate the hypothesis that adsorbate–solvent configurations sampled from MD simulations can be mapped to DFT-calculated interaction energies, thereby reducing reliance on hand-crafted descriptors. The ML framework alleviates the computational burden associated with extensive DFT interaction energy calculations required in multiscale modeling approaches and hence serves as a complementary tool for accelerating solvation modeling. By enabling rapid evaluation of solvent configurations across the adsorbates, solvent compositions, and pore environments represented in this study, this approach facilitates the high-throughput screening of solvent effects in heterogeneous catalysis.

An alternative approach to computing ensemble-averaged solvation interaction energies is to use classical molecular dynamics. This method benefits from extensive sampling but does not fully encode quantum chemical effects. A plot of $\Delta E_{sol}^{MD}$ versus $\Delta E_{sol}^{DFT}$ yields a slope of 0.51 and an MAE of 0.17 eV. For comparison, in our prior study on adsorbates at Pt(111)/water interfaces,^45^ the analogous slope and MAE were 0.76 and 0.07 eV. This comparison suggests a greater difference between classical and quantum methods in these confined systems. An interesting area for future work could be to better understand this difference, including not only the physicochemical phenomena, but also the accuracy of classical and DFT methods.

Another area of future work could be to scale up the training data, expanding the data set to include additional adsorbates, zeolite frameworks with different heteroatom identities and site compositions, and multisolvent systems. More systematically balanced adsorbate–solvent data sets would also enable rigorous leave-one-adsorbate-out and adsorbate-family grouped validation to quantify transferability to chemically unseen species. To overcome the data bottleneck in training such models, an additional promising direction is transfer learning. Future efforts could explore universal models pretrained on large-scale data sets of diverse molecular structures, followed by fine-tuning on domain-specific systems via multitask learning approaches to further improve generalization. Collectively, these advancements could enable rapid predictions within multiscale simulations and accelerate catalyst design.

## Supplementary Material



## Data Availability

All data and software necessary to reproduce the results reported in this study are publicly available at the following repository: https://github.com/getman-research-group/3dcnn-zeolite-solvation. The repository includes a machine-readable data set containing DFT-calculated adsorbate-solvent interaction energies $\left(\Delta E_{int}^{DFT}\right)$ for all systems listed in the Supporting Information, along with system identifiers and descriptive metadata (e.g., adsorbate, solvent composition, pore type, and snapshot index) consistent with the data set construction described in Section S1 of the Supporting Information. A representative voxel data set can be regenerated using the molecular simulation inputs and preprocessing scripts provided in the repository, enabling reproduction of model inputs and predictions. All scripts used for voxelization, feature generation, model training, evaluation, and figure generation are included, with documentation describing their usage. Example input files for molecular dynamics and density functional theory calculations are also provided for representative systems, enabling reproduction of the data generation workflow described in the Supporting Information. Due to file size constraints, full molecular dynamics trajectories and complete raw simulation outputs are not included in the repository but are available from the authors upon reasonable request. Voxelized representations are instead generated on demand using the provided preprocessing scripts.

## Notes

Additional computational methods, model implementation details, and performance analyses, including molecular models and data set composition, voxel representations and data augmentation, network architecture and learning curves, fold-specific results and parity plots, test-time augmentation, and spatial attribution maps (PDF)
