# Supplementary material for: Predicting Adsorbate-Solvent Interaction Energies in Zeolite Pores Using Convolutional Neural Networks with Attention Mechanisms
Source: J Chem Inf Model. 2026 Sep 30;66(19):12709–22. doi: 10.1021/acs.jcim.6c02080 (PMC13663866; doi:10.1021/acs.jcim.6c02080)
Supplement: Supplementary file 1 [file ci6c02080_si_001.pdf]

Supporting Information

for

Predicting Adsorbate-Solvent Interaction Energies in Zeolite Pores Using Convolutional Neural Networks with Attention Mechanisms

Jiexin Shi[a], Xiuting Chen[a], and Rachel B. Getman[a,*]

[a]William G. Lowrie Department of Chemical and Biomolecular Engineering, The Ohio State University, Columbus, OH 43210

* getman.11@osu.edu

TABLE OF CONTENTS

S1. MSS Method and Model Details 3
S2. Voxel Grids Representations 9
S2.1 Voxel grid representations generation 9
S2.2 Calculation of atomic features in voxel grids 10
S2.3 Data augmentation 12
S3. Convolutional neural network architecture 15
S3.1 Dual-branch feature extraction 15
S3.2 Interaction attention 16
S3.3 CNN backbone and residual architecture 18
S3.4 Regression head architecture 19
S3.5 Model architecture summary 19
S4. Training Details 21
S4.1 Evaluation metrics 21
S4.2 Learning curves 22
S5. Additional Results 23
S5.1 Performance bar plot for the simulation-system split 23
S5.2 Performance Results across Data-Split Strategies 23
S5.3 Test time augmentation (TTA) analysis 27
S5.4 Spatial importance maps 29
S6. Reference 34

S1. MSS Method and Model Details

This section provides additional details on the method used to generate the machine learning (ML) dataset. The dataset is generated using the multiscale sampling (MSS) framework developed by Zhang et al.[1–4]. The pure-water data are obtained from Chen et al.[5], while the methanol–water mixed-solvent data are newly generated following the same MSS protocols. The same Ti-FAU pore models and identical MD and DFT protocols are used across all solvent compositions.

The Ti-FAU zeolite is modeled as a 1 × 1 × 2 supercell (x = y = 24.433 Å, z = 48.866 Å, $\alpha = \beta = \gamma = 90°$), in which one framework Si atom is replaced by Ti to create the adsorption site. Two pore environments are considered: a hydrophilic pore, in which four hydroxyl groups are introduced adjacent to the Ti site by removing one Si atom from the framework, and a hydrophobic pore, in which the local framework surrounding the Ti site contains no hydroxyl groups[5].

Figure S1 illustrates the workflow from the full MD simulation cell to the localized structures extracted for voxelization. **Figure S1(a)** shows the initial simulation setup containing the zeolite–adsorbate region on the left and the bulk solvent reservoir on the right, with cell dimensions of 24.433 Å × 24.433 Å × 110 Å. **Figure S1(b)** the solvent-filled zeolite region after equilibration, with the external bulk-solvent reservoir omitted for clarity (comprising the 1 × 1 × 2 supercell, 24.433 Å × 24.433 Å × 48.866 Å). Subsequently, a local cubic region (16 Å × 16 Å × 16 Å) centered on the adsorbate center of mass (COM) is extracted from this structure, as shown in **Figure S1(c)**. This cubic geometry ensures standardized voxelization and consistent model inputs across all snapshots. **Figure S1(d)** shows the same region with the zeolite framework excluded, leaving only the adsorbate and solvent atoms for construction of the voxel representation.

Figure S1(e) shows the Ti-FAU pore environment at the adsorption site, with the solvent atoms omitted, to illustrate the confined pore geometry around the adsorption site.

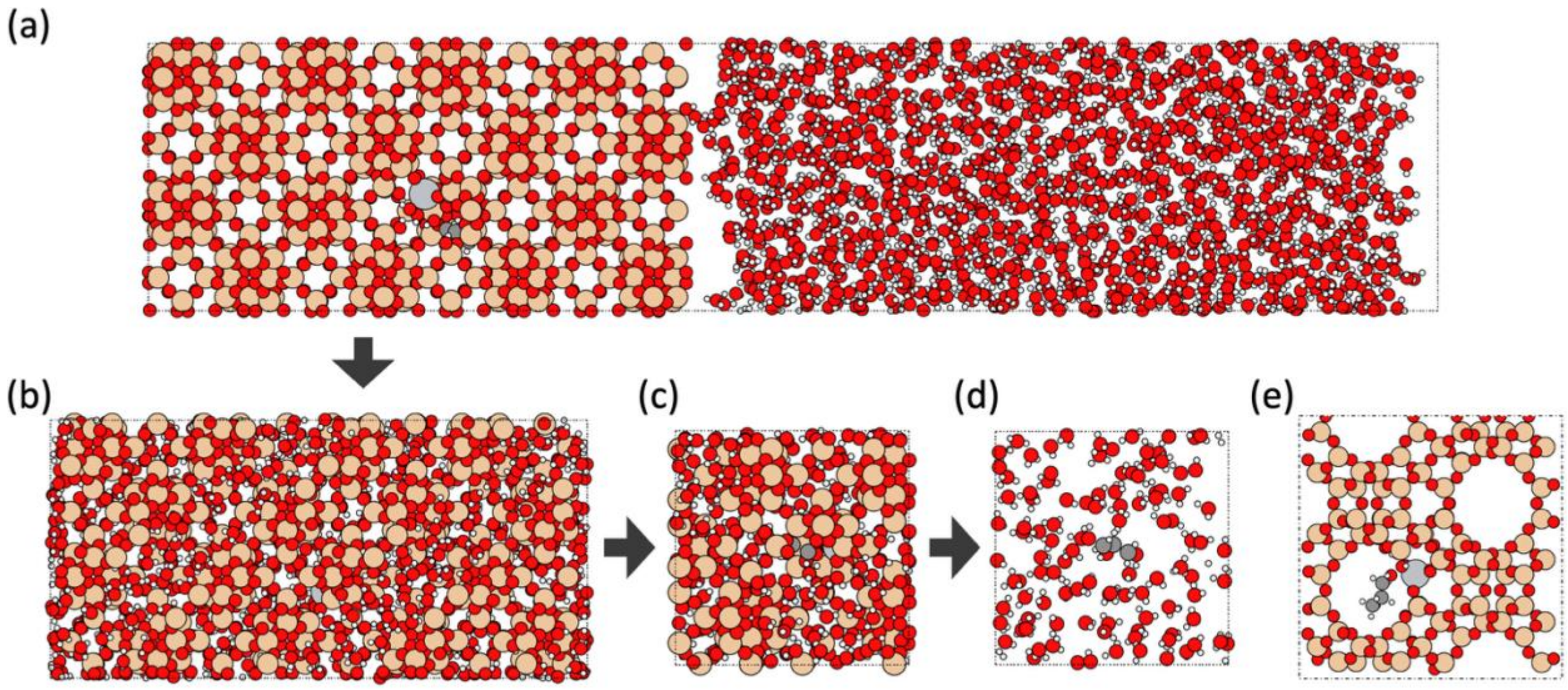

Figure S1. Visualization of the molecular systems used in this study. (a) Initial simulation cell before solvent diffusion into the zeolite pores (24.433 Å × 24.433 Å × 110 Å). (b) Solvent-filled zeolite structure with the solvent layer removed (24.433 Å × 24.433 Å × 48.866 Å). (c) Local cubic region (16 Å × 16 Å × 16 Å) centered on the adsorbate center of mass. (d) The extracted local environment retaining only the adsorbate and solvent atoms, with the zeolite framework excluded. (e) View of the Ti-FAU pore environment at the adsorption site, with solvent atoms omitted.

The adsorbates included in the dataset comprise C_1–C_3 oxygenates, specifically alcohols (methanol, ethanol, propanol, isopropanol), diols (ethylene glycol, propylene glycol, 1,3-propanediol), a triol (glycerol), a $C_3H_7O_3$ glycerol intermediate, and aldehydes (formaldehyde, acetaldehyde, and propionaldehyde). All 12 adsorbates were modeled in pure water in both the hydrophilic and hydrophobic pore models. A subset of six adsorbates—methanol, propanol, glycerol, propionaldehyde, ethylene glycol, and propylene glycol—was additionally modeled in both pore models using methanol–water mixtures with methanol mole fractions of 10%, 20%, and 50%. The mixed-solvent subset focuses on representative C_2–C_3 oxygenates spanning different functional groups, with methanol retained as a C_1 reference species. The 10% and 20% methanol

compositions represent water-rich mixtures, whereas the 50% composition provides a higher-methanol-composition comparison. The dataset is therefore not a full-factorial combination of all adsorbates, pore types, and solvent compositions. The complete dataset contains 44 pure-water systems and 12 systems at each of the three methanol mole fractions, giving 80 simulation systems in total. Of these, 40 systems contain the hydrophilic pore model and 40 contain the hydrophobic pore model. Each system contributes 10 MD snapshots with different solvent configurations for subsequent calculation of the DFT interaction energies, resulting in 800 DFT-labeled snapshots. A complete inventory of all 80 simulation systems, including the dataset configuration/system identifier, adsorbate identity, pore model, and solvent composition for each system, is provided in **Table S1**.

Table S1. Summary of the adsorbate–pore–solvent simulation systems included in the dataset.

Adsorbate	Chemical formula	Solvent environment	Hydrophilic-pore systems	Hydrophobic-pore systems	Total systems
Methanol	CH_4O	Pure water	1	1	2
Ethanol	C_2H_6O	Pure water	1	1	2
Propanol	C_3H_8O	Pure water	1	1	2
Isopropanol	C_3H_8O	Pure water	1	2	3
Formaldehyde	CH_2O	Pure water	1	1	2
Acetaldehyde	C_2H_4O	Pure water	1	1	2
Propionaldehyde	C_3H_6O	Pure water	1	1	2
Ethylene glycol	$C_2H_6O_2$	Pure water	2	2	4
Propylene glycol	$C_3H_8O_2$	Pure water	3	3	6
1,3-Propanediol	$C_3H_8O_2$	Pure water	6	4	10
Glycerol	$C_3H_8O_3$	Pure water	3	3	6
Glycerol intermediate	$C_3H_7O_3$	Pure water	1	2	3
**Pure-water subtotal**	—	**Pure water**	**22**	**22**	**44**
Methanol	CH_4O	10% methanol–water	1	1	2

Propanol	C_3H_8O	10% methanol–water	1	1	2
Propionaldehyde	C_3H_6O	10% methanol–water	1	1	2
Ethylene glycol	$C_2H_6O_2$	10% methanol–water	1	1	2
Propylene glycol	$C_3H_8O_2$	10% methanol–water	1	1	2
Glycerol	$C_3H_8O_3$	10% methanol–water	1	1	2
**10% methanol subtotal**	—	**10% methanol–water**	**6**	**6**	**12**
Methanol	CH_4O	20% methanol–water	1	1	2
Propanol	C_3H_8O	20% methanol–water	1	1	2
Propionaldehyde	C_3H_6O	20% methanol–water	1	1	2
Ethylene glycol	$C_2H_6O_2$	20% methanol–water	1	1	2
Propylene glycol	$C_3H_8O_2$	20% methanol–water	1	1	2
Glycerol	$C_3H_8O_3$	20% methanol–water	1	1	2
**20% methanol subtotal**	—	**20% methanol–water**	**6**	**6**	**12**
Methanol	CH_4O	50% methanol–water	1	1	2
Propanol	C_3H_8O	50% methanol–water	1	1	2
Propionaldehyde	C_3H_6O	50% methanol–water	1	1	2
Ethylene glycol	$C_2H_6O_2$	50% methanol–water	1	1	2
Propylene glycol	$C_3H_8O_2$	50% methanol–water	1	1	2
Glycerol	$C_3H_8O_3$	50% methanol–water	1	1	2
**50% methanol subtotal**	—	**50% methanol–water**	**6**	**6**	**12**
**Complete dataset**	—	**All solvent environments**	**40**	**40**	**80**

In all three partitioning procedures, the fold number identifies the test fold. All 10 MD snapshots belonging to a simulation system, together with their rotationally augmented representations, were assigned to the same fold. For the simulation-system split, each system was used for testing in one fold and for training in the other four folds. For the solvent-composition and pore-type splits, all systems satisfying the indicated solvent-composition or pore-type criterion were assigned to the corresponding test fold.

Table S2 Composition of the test folds under the simulation-system, solvent-composition, and pore-type splitting procedures.

Split type	Test fold	Solvent composition of test systems, n	Adsorbate composition of test systems, n	Total systems
Simulation-system split	1	Pure water (5); 10% MeOH (3); 20% MeOH (3); 50% MeOH (5)	Ethanol (1); ethylene glycol (5); glycerol (4); methanol (4); propanol (1); propylene glycol (1)	16
	2	Pure water (12); 10% MeOH (2); 20% MeOH (1); 50% MeOH (1)	1,3-Propanediol (5); ethylene glycol (2); formaldehyde (1); glycerol (1); isopropanol (2); propionaldehyde (2); propylene glycol (3)	16
	3	Pure water (8); 10% MeOH (4); 20% MeOH (3); 50% MeOH (1)	1,3-Propanediol (2); ethylene glycol (1); glycerol (2); glycerol intermediate (1); methanol (2); propanol (4); propionaldehyde (2); propylene glycol (2)	16
	4	Pure water (10); 10% MeOH (1); 20% MeOH (1); 50% MeOH (4)	1,3-Propanediol (2); acetaldehyde (2); glycerol (2); glycerol intermediate (2); isopropanol (1); methanol (2); propionaldehyde (3); propylene glycol (2)	16
	5	Pure water (9); 10% MeOH (2); 20% MeOH (4); 50% MeOH (1)	1,3-Propanediol (1); ethanol (1); ethylene glycol (2); formaldehyde (1); glycerol (3); propanol (3); propionaldehyde (1); propylene glycol (4)	16

Solvent-composition split	1	10% MeOH (12)	Ethylene glycol (2); glycerol (2); methanol (2); propanol (2); propionaldehyde (2); propylene glycol (2)	12
	2	20% MeOH (12)	Ethylene glycol (2); glycerol (2); methanol (2); propanol (2); propionaldehyde (2); propylene glycol (2)	12
	3	50% MeOH (12)	Ethylene glycol (2); glycerol (2); methanol (2); propanol (2); propionaldehyde (2); propylene glycol (2)	12
	4	Pure water (44)	1,3-Propanediol (10); acetaldehyde (2); ethanol (2); ethylene glycol (4); formaldehyde (2); glycerol (6); glycerol intermediate (3); isopropanol (3); methanol (2); propanol (2); propionaldehyde (2); propylene glycol (6)	44
Pore-type split	1	Pure water (22); 10% MeOH (6); 20% MeOH (6); 50% MeOH (6)	1,3-Propanediol (6); acetaldehyde (1); ethanol (1); ethylene glycol (5); formaldehyde (1); glycerol (6); glycerol intermediate (1); isopropanol (1); methanol (4); propanol (4); propionaldehyde (4); propylene glycol (6)	40
	2	Pure water (22); 10% MeOH (6); 20% MeOH (6); 50% MeOH (6)	1,3-Propanediol (4); acetaldehyde (1); ethanol (1); ethylene glycol (5); formaldehyde (1); glycerol (6); glycerol intermediate (2); isopropanol (2); methanol (4); propanol (4); propionaldehyde (4); propylene glycol (6)	40

S2. Voxel Grids Representations

S2.1 Voxel grid representations generation

Table S3 provides pseudocode for the voxel grid generation process, detailing the key steps: establishing spatial occupancy within adsorbate-centered grids, assigning atomic features to the corresponding voxels, spatially broadening discrete features using van der Waals radii, and normalizing continuous features.

Table S3. Voxel grid representation generation pseudocode.

```
Algorithm: Voxel grid representation generation
function CreateVoxelGrids(atomCoords, radii, features)
    for each atom A in (adsorbate ∪ solvent) do
        a ← atomCoords_A
        if A is adsorbate then
            group ← 'adsorbate'
        else if A is solvent then
            group ← 'solvent'
        end if
        a ← a - adsorbateCOM
        bin ← get_voxel_index(a)
        r_vdw ← radii_A

        for each feature f in features do
            channel ← get_channel(group, f)
            if f is discrete then
                for each voxel c within 2·r_vdw of a do
                    r ← ‖c – a‖_2
                    n ← 1 - exp(-(r_vdw/r)^12)
                    O_c,channel ← max{n, O_c,channel}
                end for
            else if f is continuous then
                O_bin,channel ← O_bin,channel + value(A, f)
            end if
        end for
    end for
    for each continuous feature f do
        maxAbsNormalize(O, channel)
    end for
end function
```

The maxAbsNormalize function applies max-absolute-value scaling (using the scikit-learn MaxAbsScaler[6]) to each continuous feature channel independently over occupied voxel positions, mapping values to the range [−1, 1]. This scaling preserves input sparsity because zero-valued voxels remain exactly zero after transformation. Furthermore, if multiple atoms fall into the same discrete voxel bin, their continuous properties are aggregated via summation prior to normalization. The van der Waals radii used for this feature broadening are $r_{vdw}(\mathrm{H}) = 1.20$ Å, $r_{vdw}(\mathrm{C}) = 1.70$ Å, and $r_{vdw}(\mathrm{O}) = 1.52$ Å. To optimize computational efficiency, the spatial broadening of these discrete features is truncated at a local distance of $2 \times r_{vdW}$ from the atomic center.

S2.2 Calculation of atomic features in voxel grids

The voxel grids incorporate 14 atomic features. All features are derived from the LAMMPS data and dump files of the classical MD production run; no additional simulations beyond the standard MSS protocol are carried out.

- **Atom Type Features (C, H, O):** Three one-hot encoded channels represent elemental identity: each atom is assigned a value of 1 in its corresponding element channel (C, H, or O) and 0 in the other channels.
- **Hydrophobic Atom:** Hydrophobic character is assigned only to adsorbate atoms using covalent connectivity inferred from element-specific interatomic-distance cutoffs. Carbon atoms are classified as hydrophobic if they have no direct oxygen neighbors; hydrogen atoms are hydrophobic if they are bonded to a hydrophobic carbon atom. By construction, all adsorbate oxygen atoms and all solvent atoms are assigned a value of 0 in this channel. Thus, this feature describes the hydrophobic character of the adsorbate rather than classifying the hydrophobicity of the solvent atoms.

- **H-bond Donor and H-bond Acceptor:** These binary features encode the potential for hydrogen bonding. An atom is labeled as a potential donor if it is a hydrogen atom covalently bonded to a single oxygen neighbor (O–H group); an atom is labeled as a potential acceptor if it is an oxygen atom. These labels reflect hydrogen-bonding capacity and are independent of whether the atom is participating in a hydrogen bond in a given snapshot.
- **Is H-bonded, Is H-bonded Donor, and Is H-bonded Acceptor:** These three snapshot-dependent features indicate actual hydrogen bond participation between adsorbate and solvent molecules in each MD snapshot. Hydrogen bonds are detected using MDAnalysis[7,8], considering both adsorbate-as-donor and solvent-as-donor scenarios. A hydrogen bond D–H···A is considered present when the following geometric criteria[9] are satisfied: The donor–acceptor distance (e.g., the $\mathrm{O_{solvent} - O_{ads}}$) < 3.5Å. When the solvent molecule is the H-bond donor, the angle formed by the solvent molecule oxygen atom, the participating solvent molecule hydrogen atom, and the adsorbate oxygen atom, (i.e., the $\mathrm{O_{solvent} - H_{solvent} \cdots O_{ads}}$ angle) > 150º. When the adsorbate is the H-bond donor, the angle formed by the adsorbate oxygen atom, the participating adsorbate hydrogen atom, and the solvent molecule oxygen atom, (i.e., the $\mathrm{O_{ads} - H_{ads} \cdots O_{solvent}}$ angle) > 150º. Donor–hydrogen pairs (i.e., the $\mathrm{O_{solvent} - H_{solvent}}$ or $\mathrm{O_{ads} - H_{ads}}$) are identified using a covalent bond distance threshold of $\mathrm{d_{D-H}}$ < 1.0Å. The geometric criteria used to detect hydrogen bonds between adsorbates and solvent molecules are illustrated in **Figure S2**.

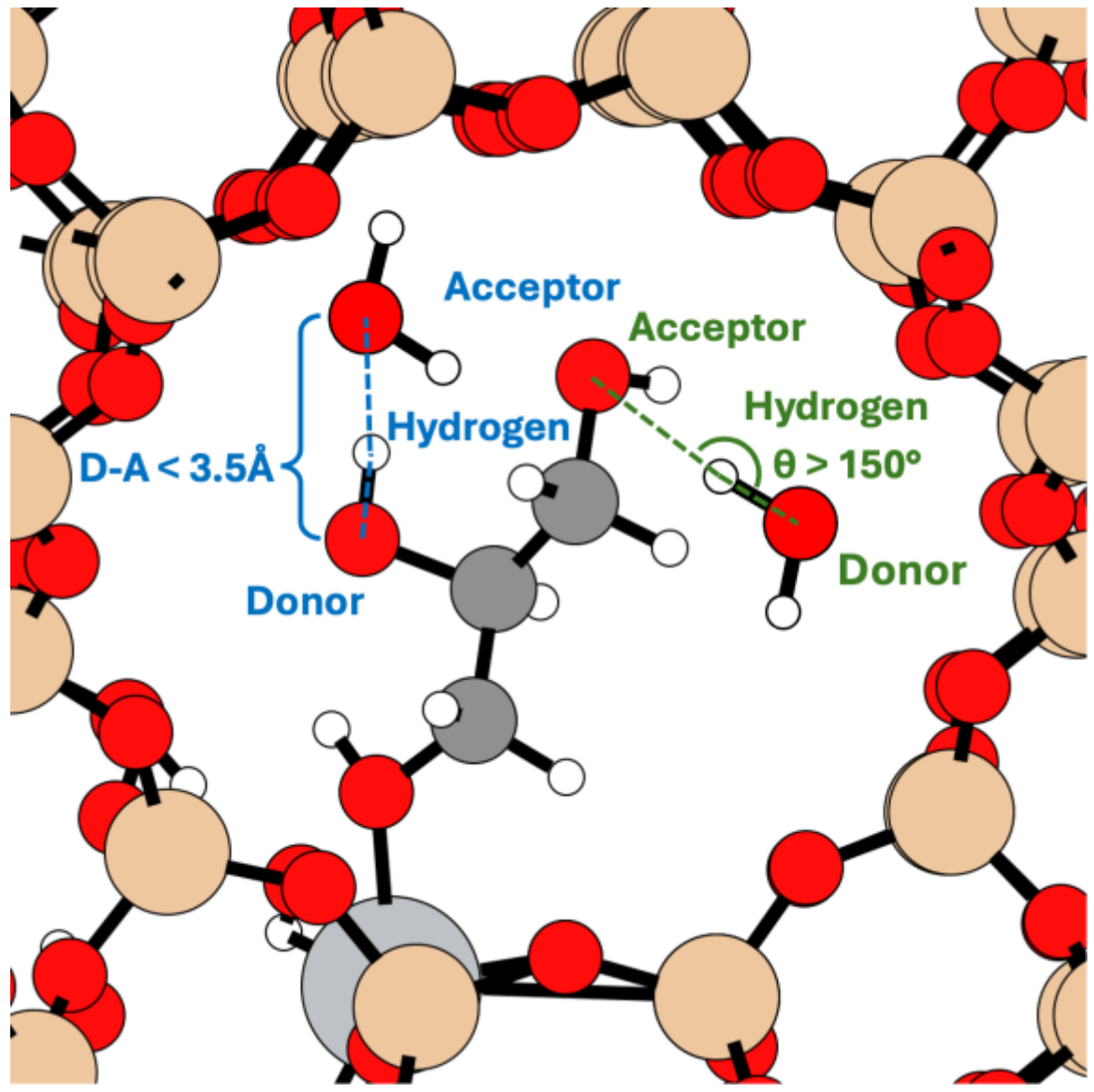

Figure S2. Geometric criteria for detecting hydrogen bonds between adsorbate and solvent molecules.

The five continuous atomic features are parsed directly from the LAMMPS data file using in-house scripts. Atomic mass, partial charge, and Lennard-Jones parameters (ε and σ) are directly retrieved from the LAMMPS data file. The valence number is defined by counting covalent bonds, where covalent connectivity is parsed from the Bonds section of the LAMMPS data file. All continuous features are then normalized per channel using max-absolute scaling over occupied voxels only, producing values in the range [−1, 1] while leaving empty voxels at zero. These features are obtained from the existing molecular-simulation topology and therefore require no additional MD or DFT calculations. Although atomic mass is closely correlated with element identity, partial charge, Lennard–Jones parameters, and valence can distinguish atoms of the same element with different force-field parameters or bonding environments.

S2.3 Data augmentation

To enhance model generalization, data augmentation is employed to generate physically equivalent grid variants. Since each 20 × 20 × 20 voxel grid is a spatial cube centered on the

adsorbate's center of mass, the complete set of 24 rotational symmetries of a cube is applied without altering the physical content of the representation. All 24 variants retain the same target interaction energy label as the original snapshot. These transformations are constructed by composing sequences of 90° rotations around the x- and y-axes. Accordingly, each of the 24 symmetry-equivalent variants is generated by applying a specific ordered sequence of these fundamental rotations, where 'x' denotes a 90° rotation around the x-axis and 'y' denotes a 90° rotation around the y-axis. For example, the sequence 'xy' corresponds to a 90° rotation around the x-axis followed by a 90° rotation around the y-axis. More complex sequences, such as 'xxxy', represent a 270° rotation around x-axis (three consecutive 90° rotations) followed by a 90° rotation around the y-axis. These combinations of x and y rotations result in 24 distinct orientations including rotations equivalent to z-axis rotations (e.g., 'xxyy' is equivalent to a 180° rotation around the z-axis). The complete set of rotation sequences employed in this work is listed in **Table S4**.

Table S4. Complete set of 24 cube rotations used for voxel grid augmentation

Rotation Name	Description
identity	No rotation (original orientation)
x	90° around x-axis
y	90° around y-axis
xx	180° around x-axis
xy	90° around x-axis, then 90° around y-axis
yx	90° around y-axis, then 90° around x-axis
yy	180° around y-axis
xxx	270° around x-axis
xxy	180° around x-axis, then 90° around y-axis
xyx	90° around x-axis, 90° around y-axis, then 90° around x-axis
xyy	90° around x-axis, then 180° around y-axis
yxx	90° around y-axis, then 180° around x-axis
yyx	180° around y-axis, then 90° around x-axis
yyy	270° around y-axis
xxxy	270° around x-axis, then 90° around y-axis
xxyx	180° around x-axis, 90° around y-axis, then 90° around x-axis
xxyy	180° around z-axis (equivalent)
xyxx	90° around x-axis, 90° around y-axis, then 180° around x-axis
xyyy	90° around x-axis, then 270° around y-axis

yxxx	90° around y-axis, then 270° around x-axis
yyyx	270° around y-axis, then 90° around x-axis
xxxyx	90° around z-axis (equivalent)
xyxxx	270° around z-axis (equivalent)
xyyyx	90° around x-axis, 270° around y-axis, then 90° around x-axis

To illustrate these transformations, **Figure S3** presents the complete set of 24 rotational variants of the voxel grid. This visualization displays a representative system comprising a methanol adsorbate in a hydrophilic Ti-FAU pore with a methanol–water solvent mixture containing a methanol mole fraction of 20%.

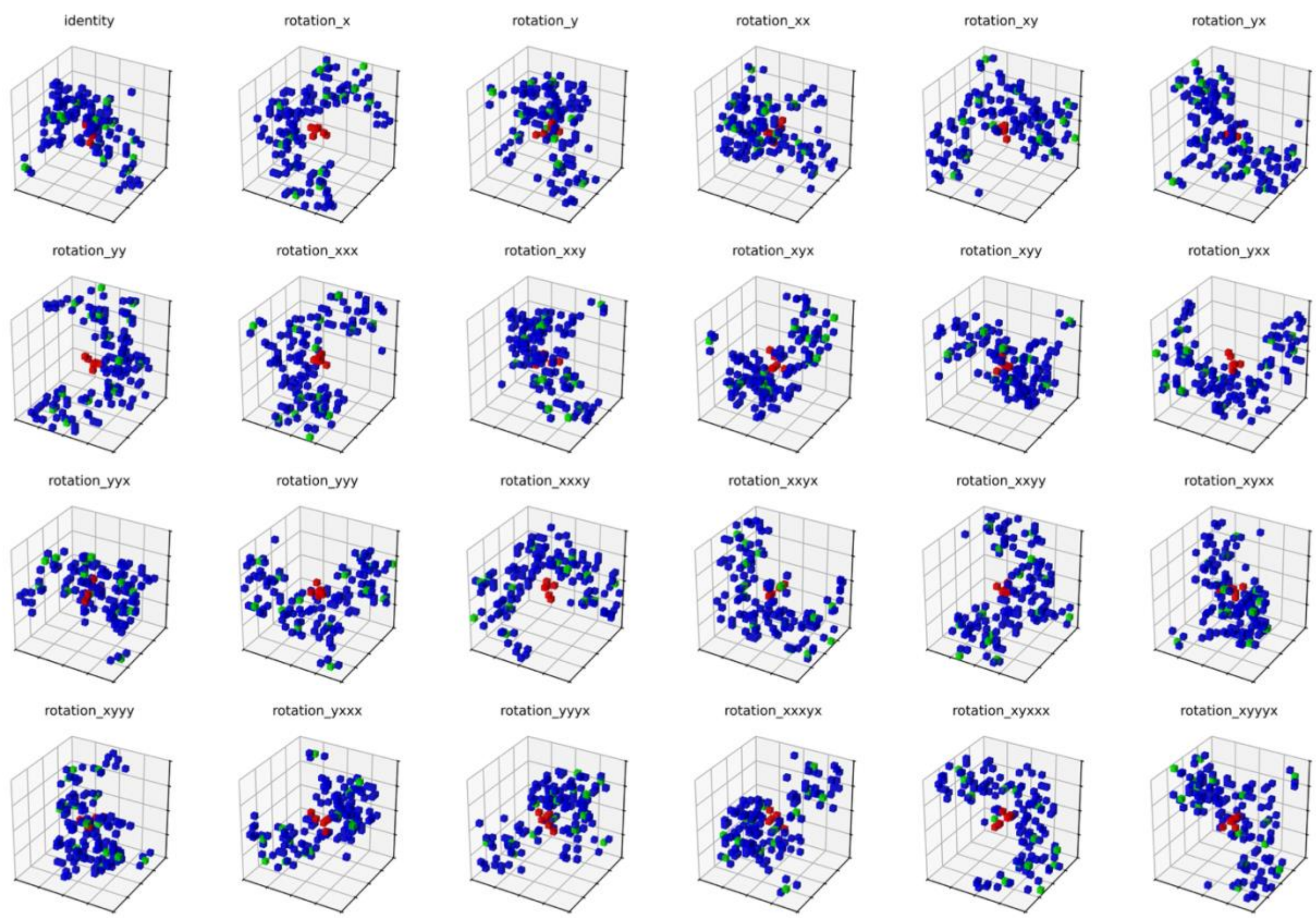

Figure S3. Visualization of the complete set of 24 rotationally augmented voxel grids for a methanol adsorbate in a hydrophilic Ti-FAU pore with a 20% methanol–water solvent mixture.

S3. Convolutional neural network architecture

The 3D-CNN model accepts input tensors of shape (batch size, 28, 20, 20, 20), where the 28 input channels correspond to 14 atomic features for each of the two molecular groups (adsorbate and solvent). Channels 0-13 encode adsorbate features and channels 14-27 encode solvent features.

S3.1 Dual-branch feature extraction

Both branch processors incorporate Convolutional Block Attention Module (CBAM)[10] blocks, which apply sequential channel and spatial attention to adaptively re-weight feature maps. For an input feature map $F \in \mathbb{R}^{C\times D\times H\times W}$, the CBAM channel attention weights are computed as:

$$M_c(F) = \sigma\left(MLP\big(AvgPool(F)\big) + MLP\big(MaxPool(F)\big)\right) \quad \text{S1}$$

where σ denotes the sigmoid activation, $AvgPool$ and $MaxPool$ are global 3D pooling operations that produce channel features in $\mathbb{R}^C$, and the multi-layer perceptron (MLP) consists of two linear layers with a bottleneck: $\mathbb{R}^C \to \mathbb{R}^{\lfloor C/r \rfloor} \to \mathbb{R}^C$, with reduction ratio r. The spatial attention map is then computed from the channel-refined feature map $F' = M_c(F) \odot F$:

$$M_s(F') = \sigma\Big(f^{k\times k\times k}([AvgPool_c(F'); MaxPool_c(F')])\Big) \quad \text{S2}$$

where $AvgPool_c$ and $MaxPool_c$ denote average and maximum pooling along the channel dimension, $[;]$ denotes concatenation, and $f^{k\times k\times k}$ is a 3D convolution with kernel size k. The final CBAM output is $M_s(F')\odot F'$.

Adsorbate Branch Architecture: The adsorbate processor employs a progressive channel expansion (14 → 20 → 26 → 32). An initial 3 × 3 × 3 convolution extracts local molecular geometry (14→20 channels), followed by a CBAM[10] attention module (Eqs. S1 and S2; reduction ratio=8, kernel size=3) applied at 20 channels. A 1 × 1 × 1 pointwise convolution (20→26

channels) performs cross-channel feature recombination, followed by a final 3 × 3 × 3 convolution (26→32 channels) for spatial feature integration. Each branch convolutional layer is followed by batch normalization and ReLU activation.

Solvent Branch Architecture: The solvent processor implements a mixed-kernel strategy. The first layer employs 5 × 5 × 5 kernels (14→32 channels) to capture distributed solvent interactions within an approximate 4.0 Å receptive field at 0.8 Å voxel resolution, followed by CBAM[10] attention (Eqs. S1 and S2; reduction ratio=6, kernel size=5) applied at 32 channels. The second layer uses 3 × 3 × 3 kernels (32→48 channels) for refined feature integration. Similarly, each branch convolutional layer incorporates batch normalization and ReLU activation.

S3.2 Interaction attention

The interaction attention module implements group-aware attention at the feature fusion point. After concatenating the adsorbate and solvent branch outputs (32 + 48 = 80 channels), a group-aware CBAM[10] attention module (reduction ratio=6, kernel size=5) is applied before convolution mixing to weight adsorbate and solvent features separately while modeling their cross-group interactions.

Group-aware channel attention: The standard CBAM channel attention (Eq. S1) processes all channels through a single shared MLP. The interaction attention module instead uses separate group-specific MLPs for the 80-channel concatenated features (group split: 32 adsorbate + 48 solvent channels). Separate MLPs process each molecular group, with bottleneck dimensions of 5 ($=\lfloor 32/6 \rfloor$) and 8 ($=\lfloor 48/6 \rfloor$) for adsorbate and solvent channels, respectively. For each group, global average-pooled and max-pooled features are processed through the same group-specific

MLP; the two outputs are summed and passed through sigmoid activation to produce per-channel attention weight vectors $a \in \mathbb{R}^{32}$ and $s \in \mathbb{R}^{48}$ for the adsorbate and solvent groups, respectively.

Cross-group interaction is governed by a learnable scalar parameter w (initialized at 0.5), passed through a sigmoid activation to yield an effective interaction factor $\sigma(w) \in (0,1)$. This encodes an asymmetric inductive bias in which the adsorbate-derived attention influences the relative importance of the surrounding solvent features. In cases where the adsorbate features are strongly weighted, the solvent channel weights are modestly amplified, allowing the model to place greater emphasis on the solvent environment when the aggregate adsorbate attention is high. The mean adsorbate channel weight $\bar{a}$ serves as a scalar summary of overall adsorbate feature importance:

$$\bar{a} = \frac{1}{N_a} \sum_{i=1}^{N_a} a_i \qquad \text{S3}$$

This scalar then multiplicatively modulates solvent weights, so that higher adsorbate importance slightly amplifies solvent attention:

$$s_{enhanced} = s \cdot (1 + \sigma(w) \cdot \bar{a}) \qquad \text{S4}$$

The final solvent weights are then computed as a weighted blend between the adsorbate-modulated and original solvent weights:

$$s_{final} = \sigma(w) \cdot s_{enhanced} + \left(1 - \sigma(w)\right) \cdot s \qquad \text{S5}$$

The residual term $\left(1 - \sigma(w)\right) \cdot s$ helps preserve the original solvent channel information and limits excessive cross-group modulation. Adsorbate channel weights are applied without cross-group modification, i.e., $a_{final} = a$. Together, these operations enable adaptive modulation of the

attention weights across the adsorbate and solvent feature groups. The combined channel attention output is $[a_{final}; s_{final}] \in \mathbb{R}^{80}$, applied elementwise along the channel dimension.

Spatial attention: The spatial attention component employs a 5 × 5 × 5 3D convolution (covering approximately 4.0 Å at 0.8 Å voxel resolution), selected to capture local spatial patterns associated with hydrogen bonding and short-range solvation structure. Average and maximum values are computed along the channel dimension, concatenated, and processed through this convolution, followed by sigmoid activation to generate spatial attention weights.

Interaction convolution layer: Following attention reweighting, the interaction module integrates the 80-channel attended features through two sequential convolutions. A 3 × 3 × 3 convolution compresses features from 80 to 64 channels while capturing spatial correlations across the attention-weighted feature maps. A subsequent 1 × 1 × 1 pointwise convolution further refines the representation to 48 channels. This two-stage compression strategy (80 → 64 → 48) provides smooth dimensionality reduction while preserving interaction information for downstream processing. Each convolutional layer is followed by batch normalization and ReLU activation.

S3.3 CNN backbone and residual architecture

The CNN backbone comprises three residual blocks based on the ResNet architecture[11], each containing two sequential 3 × 3 × 3 convolutions with batch normalization and ReLU activation. The ReLU activation following the second convolution is applied after the residual addition, consistent with the standard ResNet design. Shortcut connections with 1 × 1 × 1 convolutions and batch normalization are applied when channel dimensions change, ensuring proper residual addition across layers. No CBAM attention modules are applied within the backbone residual blocks; feature refinement relies on convolutional residual learning without attention. The channel progression follows a smooth evolution: 48 → 52 → 58 → 64. Spatial

dimension reduction is achieved through average pooling: a 2 × 2 × 2 pooling layer after the first residual block reduces the spatial resolution from 20^3 to 10^3, and another after the second block reduces it from 10^3 to 5^3. Following the third residual block, an adaptive average pooling layer compresses the spatial dimensions from 5^3 to 2^3, yielding a 512-dimensional feature vector (64 channels × 2^3) for the regression head.

S3.4 Regression head architecture

The regression head transforms the 512-dimensional flattened feature vector through a three-layer fully connected network with progressive compression: 512 → 128 → 64 → 1. Each hidden layer incorporates batch normalization and ReLU activation. This gradual compression is intended to limit abrupt information loss between high-dimensional feature extraction and scalar prediction. The final linear layer produces the predicted adsorbate–solvent interaction energy (ΔE_{int}) as a single scalar value.

S3.5 Model architecture summary

The 3D-CNN model contains approximately 882,000 learnable parameters (i.e., learnable weights and biases) distributed as follows: dual-branch feature extraction (14.6%), interaction fusion module (16.4%), CNN backbone with residual blocks (60.5%), and regression head (8.4%). The three CBAM attention modules collectively account for only ~0.5% of the total parameters (~4,100 parameters), introducing minimal parameter overhead to the model. A layer-by-layer summary of the architecture, including channel dimensions, kernel sizes, spatial resolutions, and parameter counts, is provided in **Table S5**.

Table S5. Layer-by-layer architecture summary of the 3D-CNN model.

Stage	Layer	Ch (in → out)	k	Spatial size	Parameters
Adsorbate branch	Conv3D + BN + ReLU	14 → 20	3^3	20^3	7,600
	CBAM (*r*=8, *k*=3)	20 → 20	—	20^3	134
	Conv3D + BN + ReLU	20 → 26	1^3	20^3	572
	Conv3D + BN + ReLU	26 → 32	3^3	20^3	22,528
	Subtotal				30,834
Solvent branch	Conv3D + BN + ReLU	14 → 32	5^3	20^3	56,064
	CBAM (*r*=6, *k*=5)	32 → 32	—	20^3	570
	Conv3D + BN + ReLU	32 → 48	3^3	20^3	41,568
	Subtotal				98,202
Interaction fusion	Concatenation	32+48 → 80	—	20^3	0
	CBAM (*r*=6, *k*=5)	80 → 80	—	20^3	3,419
	Conv3D + BN + ReLU	80 → 64	3^3	20^3	138,368
	Conv3D + BN + ReLU	64 → 48	1^3	20^3	3,168
	Subtotal				144,955
CNN backbone	Residual block 1	48 → 52	3^3	20^3	143,208
	Average pool	52	2^3	10^3	0
	Residual block 2	52 → 58	3^3	10^3	175,624
	Average pool	58	2^3	5^3	0
	Residual block 3	58 → 64	3^3	5^3	214,912
	Adaptive avg pool	64	—	2^3	0
	Subtotal				533,744
Regression head	FC + BN + ReLU	512 → 128	—	—	65,920
	FC + BN + ReLU	128 → 64	—	—	8,384
	FC (output)	64 → 1	—	—	65
	Subtotal				74,369
Total					882,104

S4. Training Details

S4.1 Evaluation metrics

Model performance is evaluated using three metrics: the coefficient of determination (R^2; Equation S6), mean absolute error (MAE; Equation S7) and root mean squared error (RMSE; Equation S8). These metrics compare machine learning predictions (ΔE_{int}^{ML}) against DFT-calculated reference values (ΔE_{int}^{DFT}). All metrics are computed on the original energy scale in eV after inverse transformation from the standardized label scale. R^2 quantifies the proportion of variance explained by the model, with values above 0.8 indicating good predictive ability in this work. MAE measures the average absolute deviation, providing interpretable error magnitudes in eV. RMSE penalizes larger errors more heavily than MAE through the squaring of residuals, revealing model sensitivity to relatively large prediction errors. The difference between RMSE and MAE provides a qualitative indication of the influence of larger errors, with a larger gap indicating a greater contribution from relatively large errors. These three metrics are defined as follows:

$$R^2 = 1 - \frac{\sum_{i=1}^{N}(y_i^{DFT} - y_i^{ML})^2}{\sum_{i=1}^{N}(y_i^{DFT} - \bar{y}^{DFT})^2} \quad \text{S6}$$

$$MAE = \frac{1}{N}\sum_{i=1}^{N}\left|y_i^{DFT} - y_i^{ML}\right| \quad \text{S7}$$

$$RMSE = \sqrt{\frac{1}{N}\sum_{i=1}^{N}(y_i^{DFT} - y_i^{ML})^2} \quad \text{S8}$$

where N is the number of samples, y_i^{DFT} represents the DFT-calculated reference value (i.e., ΔE_{int}^{DFT}), y_i^{ML} represents the ML model prediction (i.e., ΔE_{int}^{ML}), and $\bar{y}^{DFT}$ is the mean of the DFT reference values.

S4.2 Learning curves

The training dynamics of the 3D-CNN model are monitored by tracking the evolution of loss and gradient statistics over 200 epochs, as shown in **Figure S4**. **Figure S4(a)** displays the training and validation MSE losses computed on the standardized label scale, averaged across the available cross-validation folds at each epoch, with the shaded region indicating ±1 standard deviation. The model converges rapidly within the first ~20 epochs, after which both curves decrease steadily over the 200-epoch training period and stabilize after approximately epoch 50. Although a gap develops between the training and validation losses, the validation loss does not exhibit a sustained upward trend. **Figure S4(b)** shows the gradient norm evolution across all folds. The mean total gradient norm, computed before gradient clipping and averaged across mini-batches and folds, exhibits an overall decreasing trend without sustained spikes or divergence. The shaded region (±1σ across folds) indicates limited inter-fold variability, showing broadly similar optimization behavior across folds. The recorded gradient norms provide no evidence of gradient explosion or numerical divergence during training.

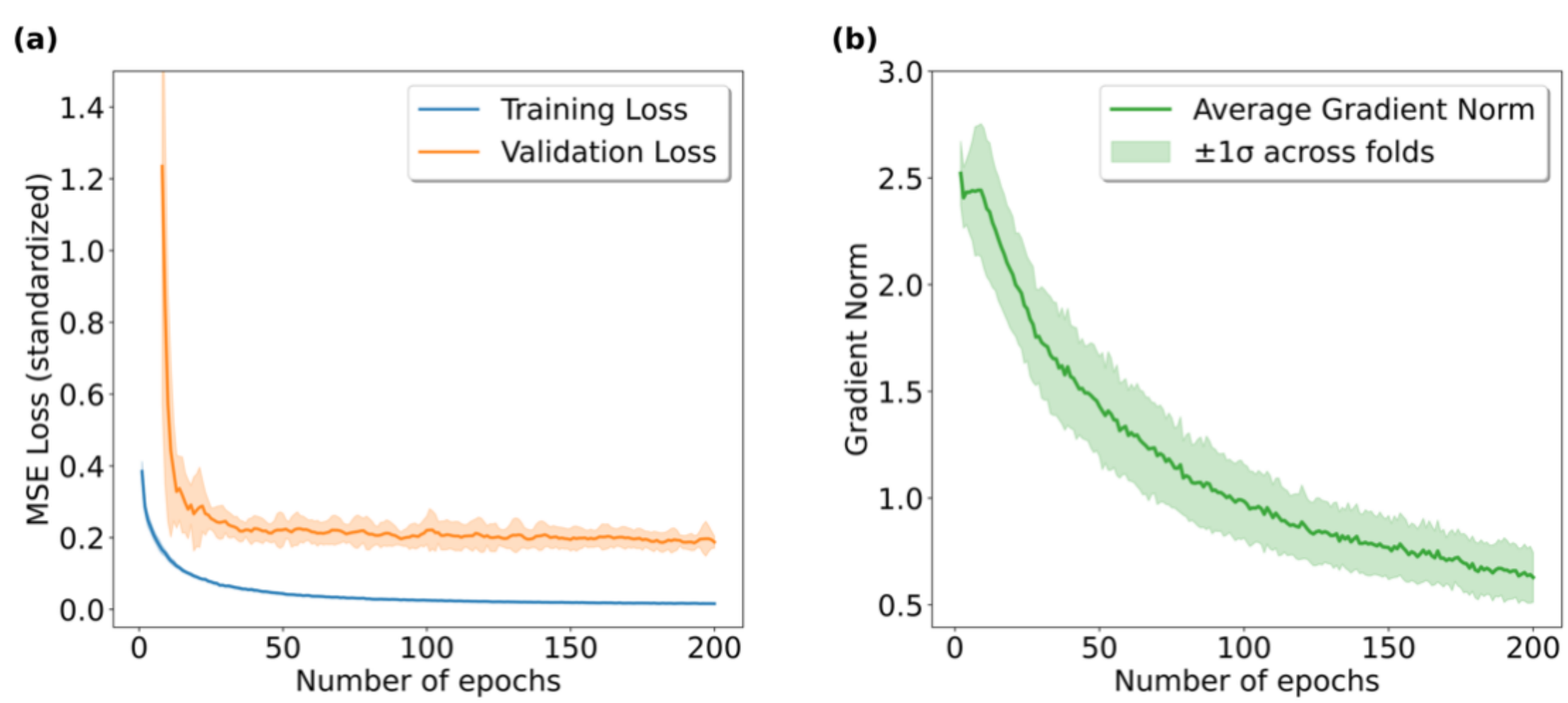

Figure S4. Training dynamics and optimization behavior of the 3D-CNN model across the five-fold cross-validation. (a) Training and validation loss curves. (b) Gradient norm evolution during training.

S5. Additional Results

S5.1 Performance bar plot for the simulation-system split

The predictive performance of the models under the primary five-fold simulation-system split is presented in **Figure S5**, which displays RMSE, MAE, and R^2 values for both the training (blue) and test (red) sets. Results are reported for predictions of the per-snapshot interaction energies ΔE_{int}^{DFT} and the ensemble-averaged solvation energies ΔE_{sol}^{DFT}. Training results are included in the bar plots for reference. The overall test-set performance (MAE = 0.083 eV for ΔE_{int} and 0.037 eV for ΔE_{sol}) indicates good predictive accuracy on the held-out simulation systems within the represented chemical domain.

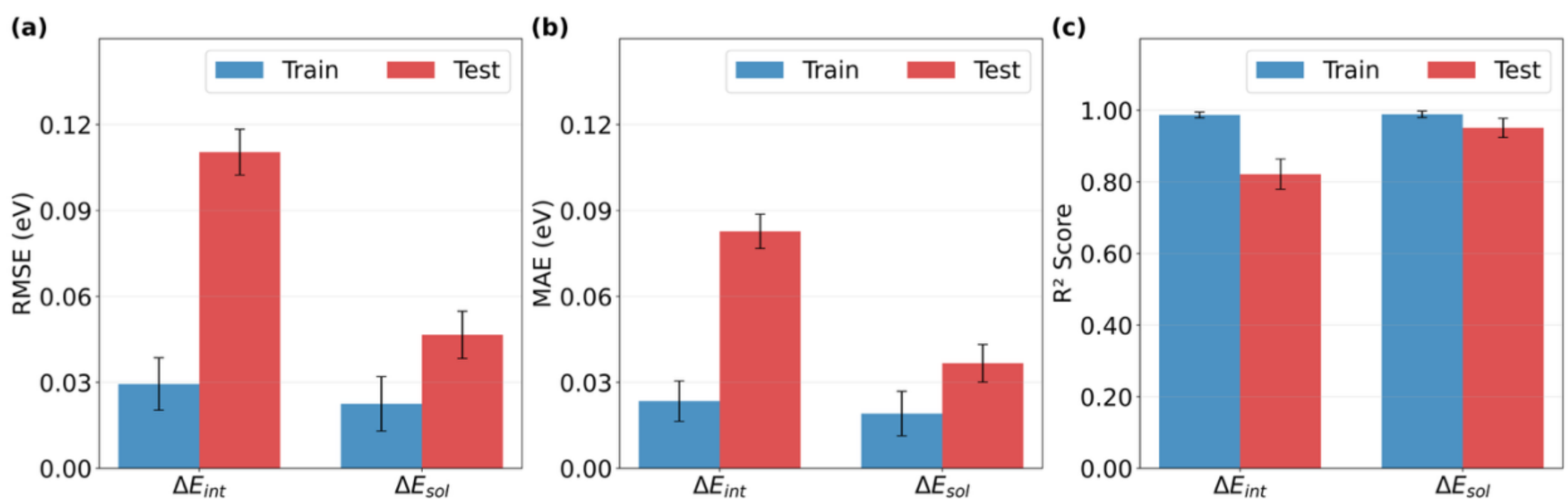

Figure S5. Performance of the 3D-CNN model in predicting ΔE_{int} and ΔE_{sol}, with RMSE, MAE, and R^2 scores. Blue and red bars represent training and test results, respectively. Error bars indicate the standard deviation across the five folds.

S5.2 Performance Results across Data-Split Strategies

This section presents detailed performance results for the simulation-system, solvent-composition, and pore-type data-split strategies. **Table S6** provides the fold-specific test-set metrics for all three strategies, enabling comparison of model performance across the held-out folds. **Figure S6** and **Figure S7** complement these quantitative results by presenting parity plots

for the solvent-composition and pore-type splits, respectively, in addition to the simulation-system parity plots presented in Figure 6 of the main text.

Table S6 reports the fold-specific test-set RMSE, MAE, and R^2 values for both ΔE_{int} and ΔE_{sol} under all three data-split strategies. The simulation-system split comprises five folds with 16 held-out systems per fold. The solvent-composition split comprises four folds, with 12 held-out systems for each methanol–water composition and 44 held-out systems for the pure-water composition. The pore-type split comprises two folds with 40 held-out systems per fold. For each strategy, the final row reports the unweighted arithmetic mean of its fold-specific metrics. Because the pure-water fold differs from the mixed-solvent folds in both test-set size and adsorbate coverage, the fold-to-fold variation under the solvent-composition split reflects these dataset characteristics in addition to the held-out solvent composition.

Table S6. Fold-specific test-set performance under the three data-split strategies. For each strategy, the final row reports the unweighted arithmetic mean of the corresponding fold-specific metrics. The value of n indicates the number of simulation systems in the test fold.

**Data-split strategy**	**Held-out test fold**	**Snapshot RMSE (eV)**	**Snapshot MAE (eV)**	**Snapshot R^2**	**System-averaged RMSE (eV)**	**System-averaged MAE (eV)**	**System-averaged R^2**
Simulation-system	Fold 1 (n = 16)	0.117	0.087	0.845	0.043	0.036	0.972
	Fold 2 (n = 16)	0.119	0.089	0.764	0.057	0.042	0.919
	Fold 3 (n = 16)	0.111	0.078	0.841	0.046	0.033	0.965
	Fold 4 (n = 16)	0.099	0.075	0.866	0.034	0.027	0.979
	Fold 5 (n = 16)	0.107	0.085	0.791	0.054	0.045	0.919
	**Unweighted mean**	**0.110**	**0.083**	**0.821**	**0.047**	**0.037**	**0.951**
Solvent composition	10% methanol–water (n = 12)	0.117	0.085	0.813	0.066	0.048	0.926
	20% methanol–water (n = 12)	0.091	0.072	0.858	0.028	0.023	0.981
	50% methanol–water (n = 12)	0.084	0.062	0.855	0.017	0.013	0.991

	Pure water (n = 44)	0.146	0.113	0.682	0.078	0.062	0.869
	**Unweighted mean**	**0.110**	**0.083**	**0.802**	**0.048**	**0.036**	**0.942**
Pore type	Hydrophilic (n = 40)	0.139	0.109	0.746	0.078	0.067	0.897
	Hydrophobic (n = 40)	0.128	0.102	0.717	0.065	0.051	0.886
	**Unweighted mean**	**0.133**	**0.106**	**0.732**	**0.071**	**0.059**	**0.891**

Figure S6 presents the parity plots obtained from the solvent-based 4-fold data-split strategy, in which the model is trained on three solvent compositions and tested on the held-out fourth. This approach evaluates the model's ability to predict interaction energetics for adsorbates in a solvent composition excluded from the corresponding training fold. **Figure S6(a)** shows per-snapshot ΔE_{int}^{ML} vs. ΔE_{int}^{DFT} (test MAE = 0.083 eV, R^2 = 0.80). **Figure S6(b)** presents the ensemble-averaged ΔE_{sol}^{ML} vs. ΔE_{sol}^{DFT} (test MAE = 0.036 eV, R^2 = 0.94). **Figure S6(c)** compares ML-predicted ΔE_{sol}^{ML} and MD-calculated ΔE_{sol}^{MD} values against DFT reference values ΔE_{sol}^{DFT}. In (a) and (b), blue triangles and red circles represent training and test data, respectively; red-shaded regions indicate the 95% confidence bands around the regression lines fitted to the test data. In (c), red circles and green triangles represent ML-predicted and MD-calculated values, respectively. These metrics are comparable to those of the primary simulation-system split, supporting the model's ability to generalize across the solvent compositions considered in this work.

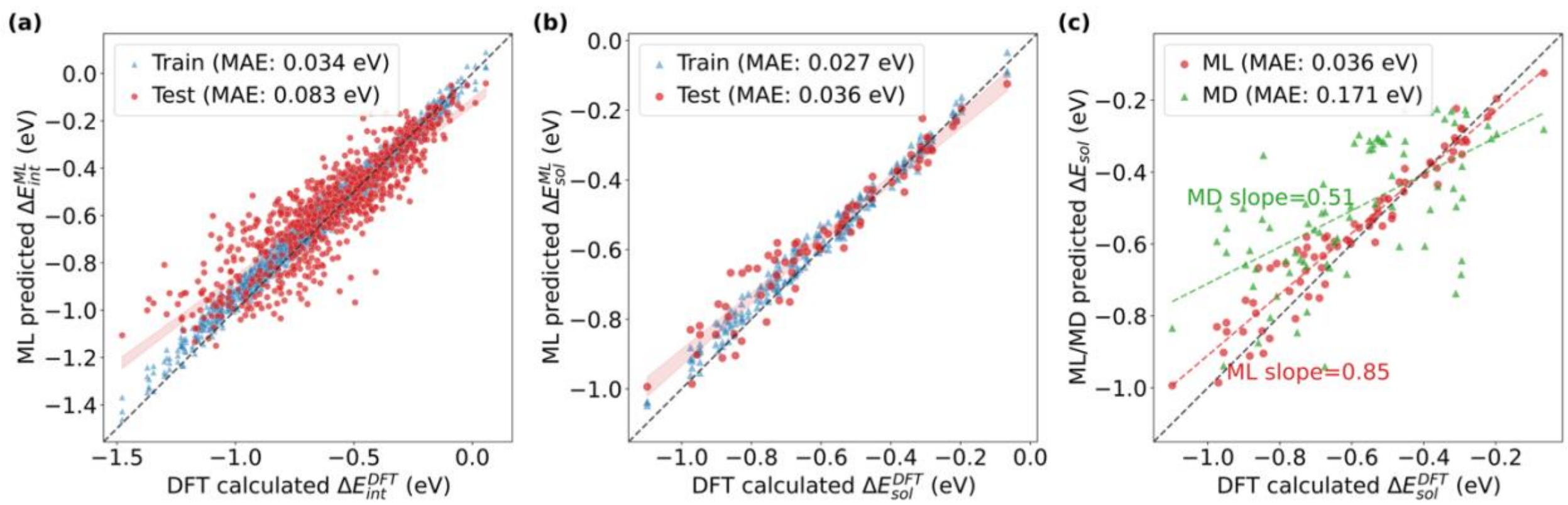

Figure S6. Parity plots for the solvent-based data-split strategy. (a) Individual snapshot predictions of ΔE_{int}^{ML} vs. ΔE_{int}^{DFT}. (b) Ensemble-averaged solvation energies ΔE_{sol}^{ML} vs. ΔE_{sol}^{DFT}. (c) ML and MD predictions compared against DFT reference values for ΔE_{sol}.

Figure S7 presents the parity plots for the pore-type-based 2-fold data-split strategy, where the model is trained on data from one pore type (e.g., hydrophilic) and tested on the other (e.g., hydrophobic). This approach evaluates the model's ability to predict interaction energetics across zeolite pores with different hydrophilicities. **Figure S7(a)** shows per-snapshot ΔE_{int}^{ML} vs. ΔE_{int}^{DFT} (test MAE = 0.106 eV, $R^2 = 0.73$). **Figure S7(b)** presents the ensemble-averaged ΔE_{sol}^{ML} vs. ΔE_{sol}^{DFT} (test MAE = 0.059 eV, $R^2 = 0.89$). **Figure S7(c)** compares ML-predicted ΔE_{sol}^{ML} and MD-calculated ΔE_{sol}^{MD} values against DFT reference values ΔE_{sol}^{DFT}. In (a) and (b), blue triangles and red circles represent training and test data, respectively; red-shaded regions indicate the 95% confidence bands around the regression lines fitted to the test data. In (c), red circles and green triangles represent ML-predicted and MD-calculated values, respectively. The slightly higher errors relative to the other splits are expected given that only two folds are available, restricting each training set to 50% of the data. Nevertheless, the ensemble-averaged R^2 of 0.89 shows that useful predictive performance is retained when transferring between the two pore types.

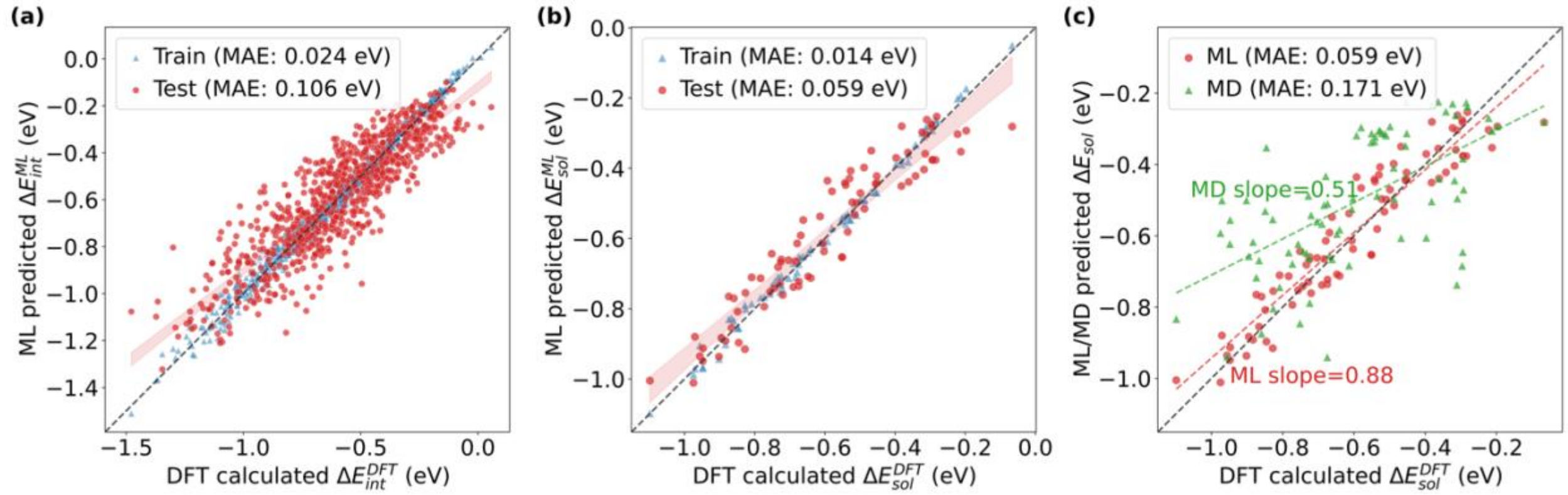

Figure S7. Parity plots for the pore-type-based data-split strategy. (a) Individual snapshot predictions of ΔE_{int}^{ML} vs. ΔE_{int}^{DFT}. (b) Ensemble-averaged solvation energies: ΔE_{sol}^{ML} vs. ΔE_{sol}^{DFT}. (c) ML-predicted and MD-calculated values compared against DFT reference values for ΔE_{sol}.

S5.3 Test time augmentation (TTA) analysis

This subsection evaluates the rotational consistency of the 3D-CNN model by comparing test performance with and without test-time augmentation (TTA) under the five-fold simulation-system split. In the TTA approach, predictions from the 24 rotated variants of each test voxel grid are averaged to obtain a single ΔE_{int}^{ML} value per snapshot. In the non-TTA approach, predictions are made directly from the original, non-augmented voxel grids.

Across all test snapshots, the mean standard deviation of the predictions across the 24 rotated variants averages 0.024 eV, which is substantially smaller than the overall prediction error, indicating that orientation-dependent prediction variability is modest relative to the overall prediction error. At the ensemble-averaged ΔE_{sol} level, the performance metrics are nearly identical: the TTA approach yields an RMSE of 0.047 eV and an MAE of 0.037 eV, compared to 0.048 eV and 0.037 eV for the non-TTA approach. This near equivalence indicates that the model predictions have limited sensitivity to input orientation.

Figure S8 provides a comprehensive comparison of model performance with and without TTA. **Figure S8(a)-(c)** show results with TTA: (a) ΔE_{int} predictions for all rotational variants, with each test snapshot represented by the mean and standard deviation of its 24 predictions; (b) per-snapshot ΔE_{int} predictions obtained by averaging the 24 rotational variants for both the training and test sets, shown without error bars; and (c) ensemble-averaged ΔE_{sol}, where the rotation-averaged predictions are further averaged over 10 snapshots per system. **Figure S8(d)-(e)** display corresponding results without TTA using only the original voxel grids: (d) predictions of ΔE_{int} on the original voxel grids for direct comparison with (b), and (e) ensemble-averaged ΔE_{sol} without TTA for comparison with (c). **Figure S8(f)** provides a quantitative performance comparison, showing the test RMSE and MAE values for ΔE_{sol} predictions with and without TTA. The small per-snapshot standard deviations in (a) and the nearly identical ΔE_{sol} error metrics in (f) together support the approximate rotational consistency of the model.

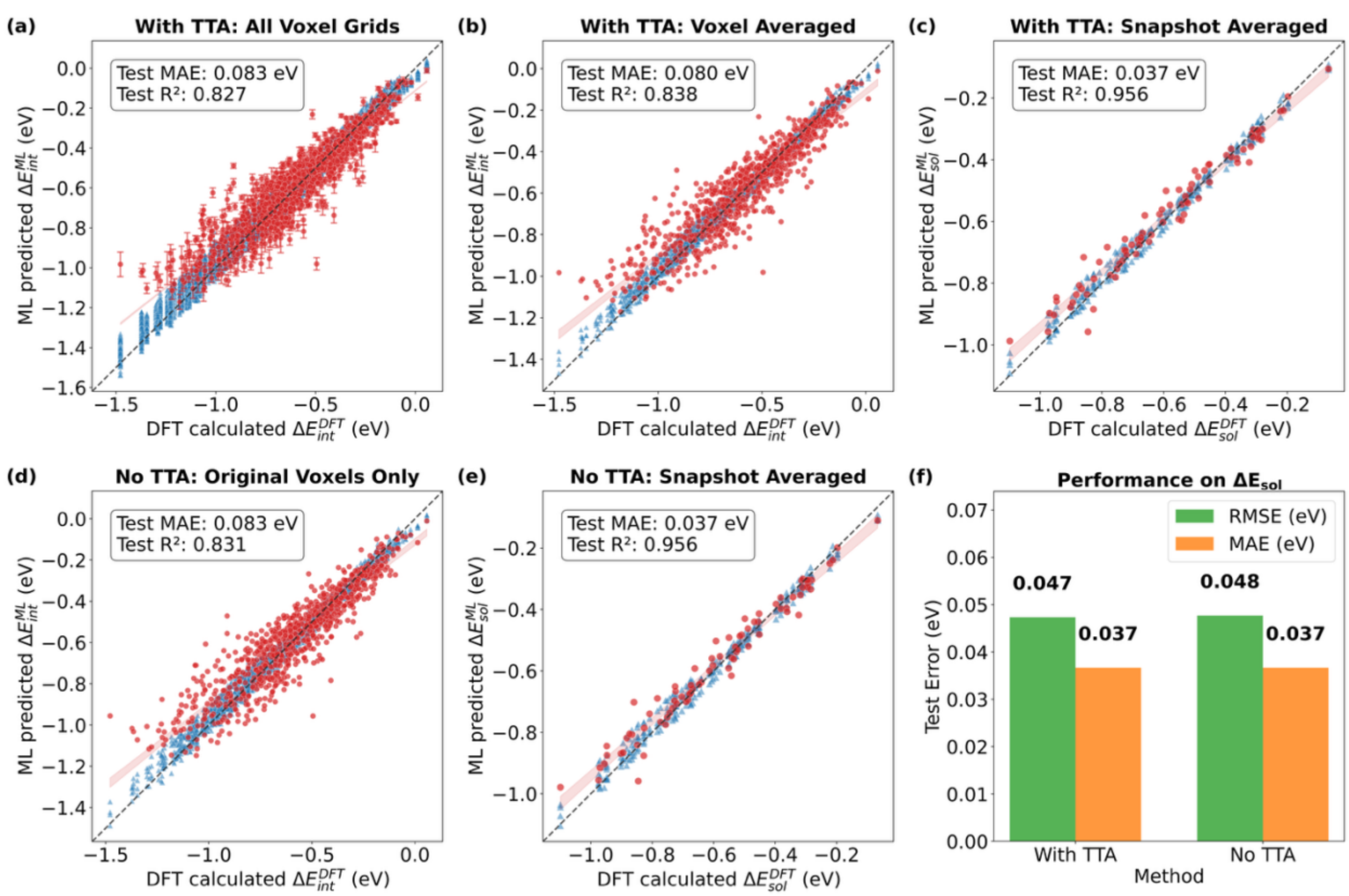

Figure S8. Comparison of test-time augmentation (TTA) effects on model performance under the five-fold simulation-system split. (a) Predictions of ΔE_{int} averaged across 24 rotated variants with error bars indicating standard deviation. (b) Per-snapshot ΔE_{int}^{ML} vs. ΔE_{int}^{DFT}, (c) Ensemble-averaged predictions ΔE_{sol}^{ML} vs. ΔE_{sol}^{DFT}. (d) Predictions on original non-augmented voxel grids ΔE_{int}^{ML} vs. ΔE_{int}^{DFT}, (e) Ensemble-averaged predictions without TTA ΔE_{sol}^{ML} vs. ΔE_{sol}^{DFT}. (f) Comparison of test RMSE and MAE values for ΔE_{sol}^{ML} predictions with and without TTA.

S5.4 Spatial importance maps

To interpret the spatial patterns learned by the 3D-CNN model and identify which spatial regions most influence the model output, we compute attribution maps of the 3D-CNN architecture using Captum[12], an open-source model interpretability library for PyTorch[13]. These analyses complement the CBAM spatial attention heatmaps shown in the main text (Figure 7), which visualize the learned attention weights within the three analyzed attention modules; by contrast, the gradient-based attributions presented here quantify how each input voxel or intermediate activation contributes to the predicted output.

Spatial attribution methods are built on the integrated gradients framework, which attributes the model output $f(\mathbf{x})$ to each feature by integrating the model's gradients along a straight-line path from a reference baseline to the actual input:

$$IG_i(\mathbf{x}) = (x_i - x_i') \int_0^1 \frac{\partial f\big(\mathbf{x}' + \alpha(\mathbf{x} - \mathbf{x}')\big)}{\partial x_i} d\alpha \qquad \text{S9}$$

where $f(\mathbf{x})$ denotes the scalar output of the trained network on the standardized target scale, $\mathbf{x}$ is the input voxel grid, $\mathbf{x}'$ is the baseline (set to zero), x_i denotes the i-th input feature (i.e., a specific channel at a specific voxel position), and $\alpha \in [0,1]$ is the interpolation parameter.

At the input voxel layer, the integrated gradients method is applied directly to compute an attribution score for every channel–voxel input value, quantifying its contribution to the model output relative to the zero baseline. The integral is approximated via Gauss–Legendre quadrature

with 100 steps. At the intermediate-layer outputs, the layer conductance method, which extends the integrated gradients framework to intermediate layers by decomposing the total input-to-output attribution through hidden neurons along a baseline-to-input interpolation path, is applied to the activations of the adsorbate processor, solvent processor, and interaction convolution layer. The absolute values of the resulting multi-channel attributions are averaged across the learned feature channels to yield a spatial importance map. This provides a complementary view of which spatial regions in the learned intermediate representations most influence the final prediction. At both levels, attributions are computed independently for each fold in which the sample appears in the training set and then averaged to produce ensemble-averaged importance maps. All maps shown below correspond to the same representative snapshot used for the attention analysis in the main text: propylene glycol in a hydrophilic FAU zeolite pore with a pure water solvent environment.

Figure S9 displays 2D spatial attribution maps from the input voxel grid for the adsorbate (top row) and solvent (bottom row) branches. Absolute attribution magnitudes are first averaged across the 14 feature channels within each branch to yield a 3D spatial importance volume, which is then projected onto three orthogonal planes by averaging along each spatial axis. The adsorbate branch exhibits localized importance around the central region of the voxel grid, where the adsorbate molecule is positioned, while the solvent branch shows more spatially extended patterns, consistent with the distribution of surrounding solvent molecules.

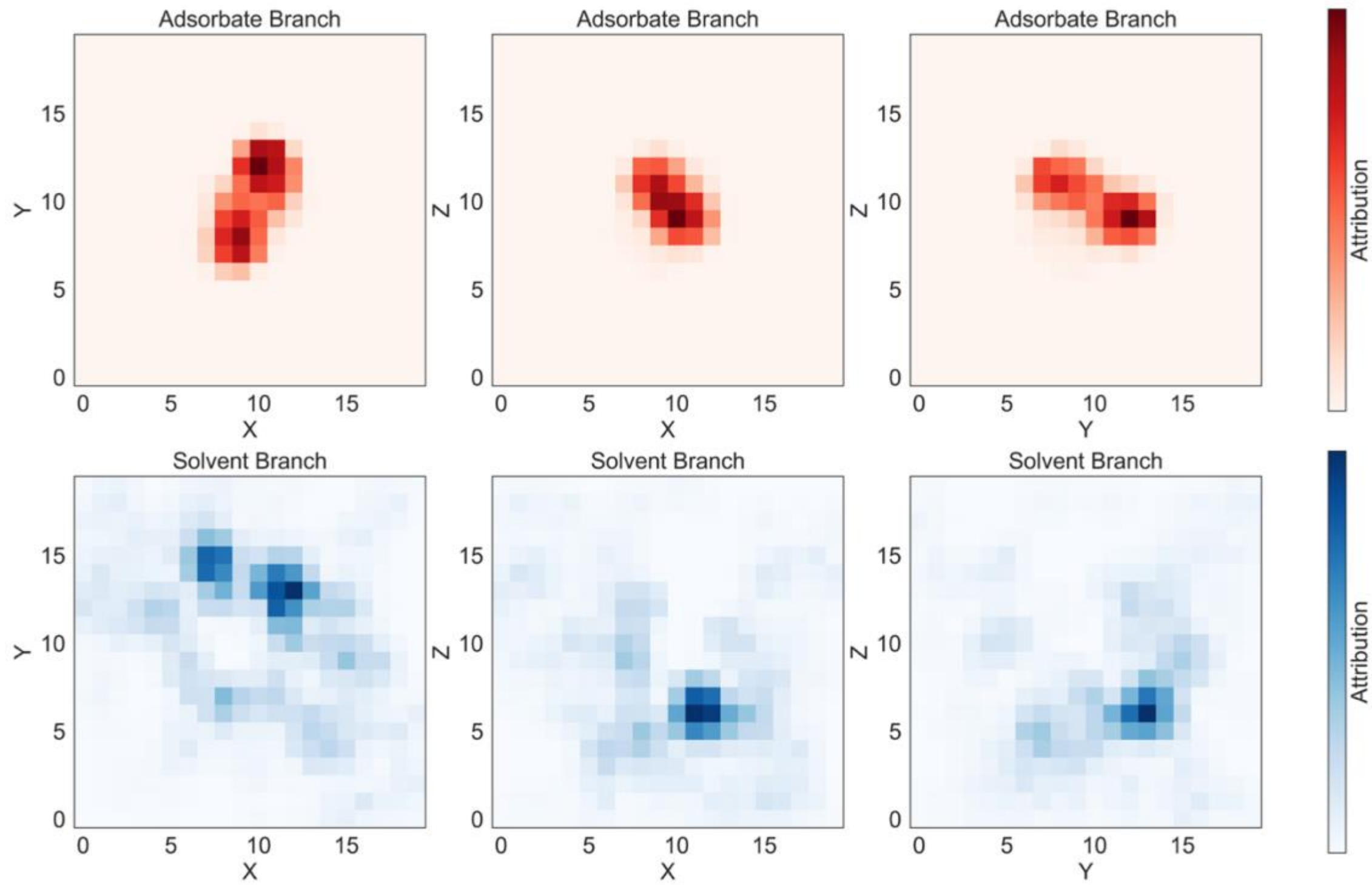

Figure S9. 2D spatial attribution maps from the input voxel grid. The adsorbate branch (top) and solvent branch (bottom) show three orthogonal projections: XY plane (left), XZ plane (middle), and YZ plane (right). Color intensity indicates the mean absolute integrated-gradients attribution across feature channels.

Figure S10 presents spatial attribution maps from the adsorbate processor, solvent processor, and interaction fusion layer, obtained via the layer conductance method described above. The 2D views are obtained by extracting central slices along each axis (for even-sized grids, the two central slices are averaged). The intermediate-layer maps exhibit broader and more continuous spatial importance patterns compared to the input-layer maps (**Figure S9**), consistent with the expanded receptive fields of the convolutional layers.

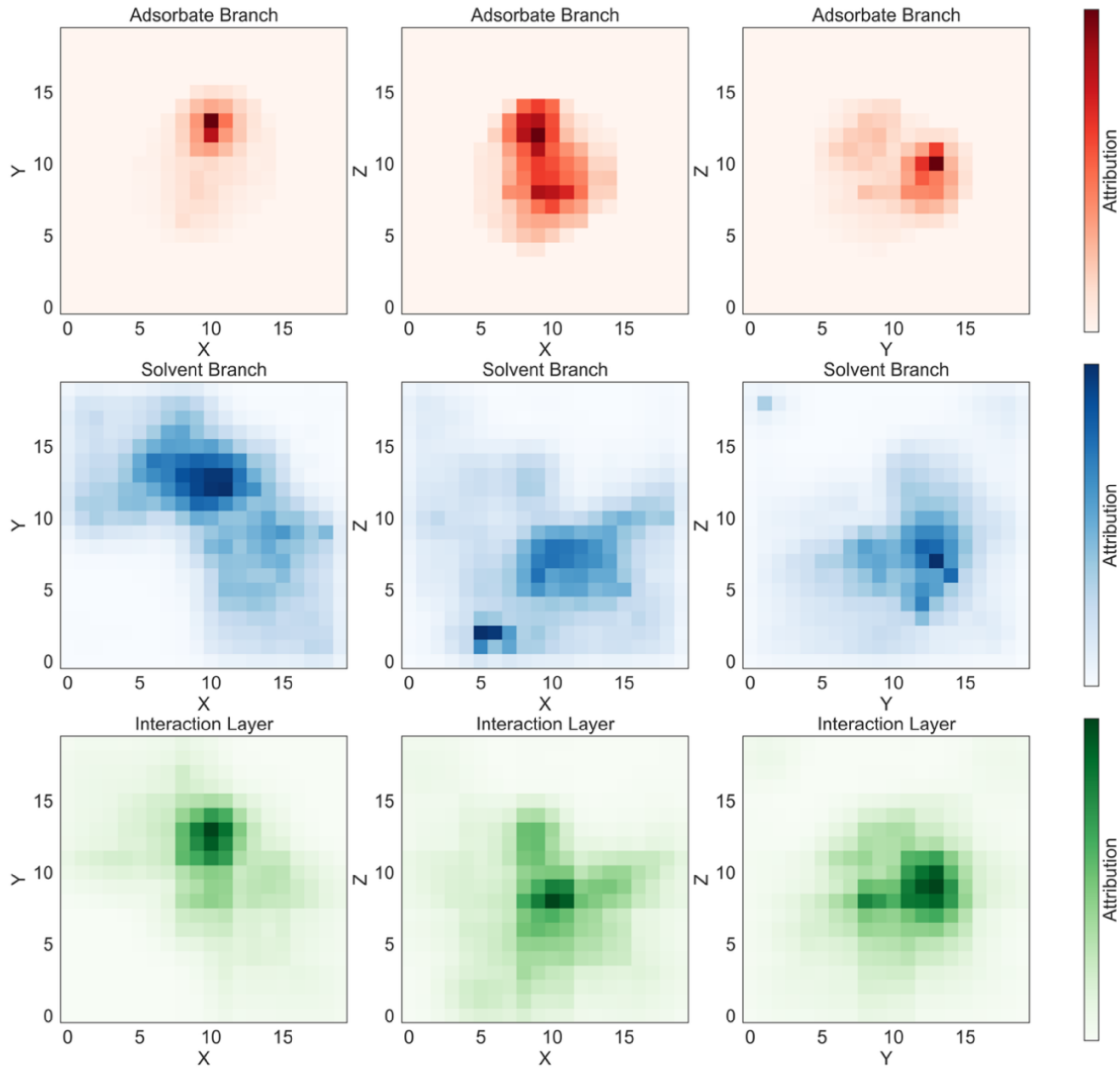

Figure S10. 2D spatial attribution maps from processor output layers. Rows correspond to the adsorbate processor (top), solvent processor (middle), and interaction layer (bottom). Columns show views along the XY plane (left), XZ plane (middle), and YZ plane (right). Color intensity represents the mean absolute layer-conductance magnitude across learned feature channels.

To complement the 2D projections, **Figure S11** displays three-dimensional spatial attribution maps at the input voxel layer for the adsorbate and solvent branches, with each voxel colored according to its normalized attribution magnitude (0–1 scale). Low-attribution voxels are rendered transparent. The adsorbate branch shows concentrated high-attribution voxels near the center of the grid, while high-attribution solvent voxels are distributed more broadly.

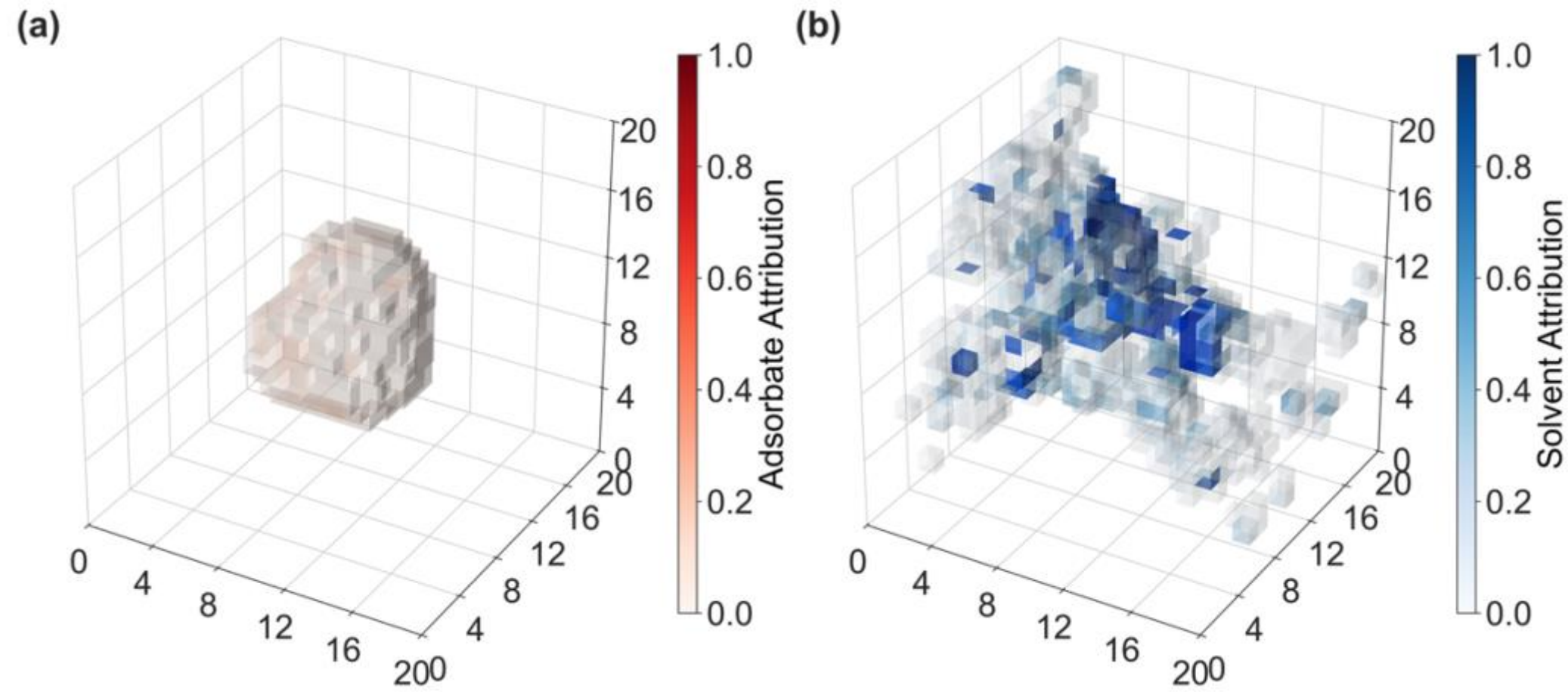

Figure S11. Three-dimensional spatial attribution maps from the input voxel layer. (a) Adsorbate branch. (b) Solvent branch. Color intensity indicates normalized mean absolute integrated-gradients attribution.

S6. Reference

(1) Zhang, X.; DeFever, R. S.; Sarupria, S.; Getman, R. B. Free Energies of Catalytic Species Adsorbed to Pt(111) Surfaces under Liquid Solvent Calculated Using Classical and Quantum Approaches. *J. Chem. Inf. Model.* **2019**, *59* (5), 2190–2198. https://doi.org/10.1021/acs.jcim.9b00089

(2) Bodenschatz, C. J.; Zhang, X.; Xie, T.; Arvay, J.; Sarupria, S.; Getman, R. B. Multiscale Sampling of a Heterogeneous Water/Metal Catalyst Interface Using Density Functional Theory and Force-Field Molecular Dynamics. *JoVE J. Vis. Exp.* **2019**, No. 146, e59284. https://doi.org/10.3791/59284

(3) Zhang, X. Development of Improved Models for Simulating Adsorption Kinetics and Solvation Free Energies of Catalytic Species at Pt(111)/Liquid Water Interfaces, Clemson University, 2021.

(4) Garcia Carcamo, R. A.; Zhang, X.; Estejab, A.; Zhou, J.; Hare, B. J.; Sievers, C.; Sarupria, S.; Getman, R. B. Differences in Solvation Thermodynamics of Oxygenates at Pt/Al2O3 Perimeter versus Pt(111) Terrace Sites. *iScience* **2023**, *26* (2), 105980. https://doi.org/10.1016/j.isci.2023.105980

(5) Chen, X.; Getman, R. B. The Significant Differences in Solvation Thermodynamics of C1–C3 Oxygenates in Hydrophilic versus Hydrophobic Pores of a Hydrophilic Ti-FAU Zeolite Model. *J. Phys. Chem. C* **2024**, *128* (45), 19367–19379. https://doi.org/10.1021/acs.jpcc.4c04596

(6) Pedregosa, F.; Varoquaux, G.; Gramfort, A.; Michel, V.; Thirion, B.; Grisel, O.; Blondel, M.; Prettenhofer, P.; Weiss, R.; Dubourg, V.; Vanderplas, J.; Passos, A.; Cournapeau, D.; Brucher,

M.; Perrot, M.; Duchesnay, É. Scikit-Learn: Machine Learning in Python. *J. Mach. Learn. Res.* **2011**, *12* (85), 2825–2830.

(7) Gowers, R. J.; Linke, M.; Barnoud, J.; Reddy, T. J. E.; Melo, M. N.; Seyler, S. L.; Domański, J.; Dotson, D. L.; Buchoux, S.; Kenney, I. M.; Beckstein, O. MDAnalysis: A Python Package for the Rapid Analysis of Molecular Dynamics Simulations. *Proc. 15th Python Sci. Conf.* **2016**, 98–105. https://doi.org/10.25080/Majora-629e541a-00e

(8) Michaud-Agrawal, N.; Denning, E. J.; Woolf, T. B.; Beckstein, O. MDAnalysis: A Toolkit for the Analysis of Molecular Dynamics Simulations. *J. Comput. Chem.* **2011**, *32* (10), 2319–2327. https://doi.org/10.1002/jcc.21787

(9) Luzar, A.; Chandler, D. Hydrogen-Bond Kinetics in Liquid Water. *Nature* **1996**, *379* (6560), 55–57. https://doi.org/10.1038/379055a0

(10) Woo, S.; Park, J.; Lee, J.-Y.; Kweon, I. S. CBAM: Convolutional Block Attention Module. In *Computer Vision – ECCV 2018*; Ferrari, V., Hebert, M., Sminchisescu, C., Weiss, Y., Eds.; Springer International Publishing: Cham, 2018; pp 3–19. https://doi.org/10.1007/978-3-030-01234-2_1

(11) He, K.; Zhang, X.; Ren, S.; Sun, J. Deep Residual Learning for Image Recognition. In *2016 IEEE Conference on Computer Vision and Pattern Recognition (CVPR)*; 2016; pp 770–778. https://doi.org/10.1109/CVPR.2016.90

(12) Kokhlikyan, N.; Miglani, V.; Martin, M.; Wang, E.; Alsallakh, B.; Reynolds, J.; Melnikov, A.; Kliushkina, N.; Araya, C.; Yan, S.; Reblitz-Richardson, O. Captum: A Unified and Generic Model Interpretability Library for PyTorch. arXiv September 16, 2020. https://doi.org/10.48550/arXiv.2009.07896

(13) Paszke, A.; Gross, S.; Massa, F.; Lerer, A.; Bradbury, J.; Chanan, G.; Killeen, T.; Lin, Z.; Gimelshein, N.; Antiga, L.; Desmaison, A.; Köpf, A.; Yang, E.; DeVito, Z.; Raison, M.; Tejani, A.; Chilamkurthy, S.; Steiner, B.; Fang, L.; Bai, J.; Chintala, S. PyTorch: An Imperative Style, High-Performance Deep Learning Library. arXiv December 3, 2019. https://doi.org/10.48550/arXiv.1912.01703
